# Ensemble based high performance deep learning models for fake news detection

**DOI:** 10.1038/s41598-024-76286-0

**Published:** 2024-11-04

**Authors:** Mohammed E.Almandouh, Mohammed F. Alrahmawy, Mohamed Eisa, Mohamed Elhoseny, A. S. Tolba

**Affiliations:** 1 Portsaid University, Portsaid, Egypt; 2 Mansoura University, Mansoura, Egypt; 3 New Mansoura University, New Mansoura, Egypt; 4 University of Economics and Human Sciences, Warsaw, Poland; 5 University of Sharjah, Sharjah, United Arab Emirates; 6 New Heliopolis Institute for Engineering & Automotive and Energy Technologies, New Heliopolis, Egypt

**Keywords:** Ensemble Learning, Deep Learning, Fake News Detection, FastText, Mathematics and computing, Computer science

## Abstract

Social media has emerged as a dominant platform where individuals freely share opinions and communicate globally. Its role in disseminating news worldwide is significant due to its easy accessibility. However, the increase in the use of these platforms presents severe risks for potentially misleading people. Our research aims to investigate different techniques within machine learning, deep learning, and ensemble learning frameworks in Arabic fake news detection. We integrated FastText word embeddings with various machine learning and deep learning methods. We then leveraged advanced transformer-based models, including BERT, XLNet, and RoBERTa, optimizing their performance through careful hyperparameter tuning. The research methodology involves utilizing two Arabic news article datasets, AFND and ARABICFAKETWEETS datasets, categorized into fake and real subsets and applying comprehensive preprocessing techniques to the text data. Four hybrid deep learning models are presented: CNN-LSTM, RNN-CNN, RNN-LSTM, and Bi-GRU-Bi-LSTM. The Bi-GRU-Bi-LSTM model demonstrated superior performance regarding the F1 score, accuracy, and loss metrics. The precision, recall, F1 score, and accuracy of the hybrid Bi-GRU-Bi-LSTM model on the AFND Dataset are 0.97, 0.97, 0.98, and 0.98, and on the ARABICFAKETWEETS dataset are 0.98, 0.98, 0.99, and 0.99 respectively. The study’s primary conclusion is that when spotting fake news in Arabic, the Bi-GRU-Bi-LSTM model outperforms other models by a significant margin. It significantly aids the global fight against false information by setting the stage for future research to expand fake news detection to multiple languages.

## Introduction

Digital platforms such as social media, online forums, and websites have overtaken traditional media as the primary sources of information ^1^. This shift signifies a substantial transformation in how we seek out and interact with information ^2^. Social media’s appeal lies in its unrestricted expression and immediate access to information, making it particularly popular among younger demographic groups. However, the ease of engagement and sharing on these platforms has also led to the rapid spread of misinformation, including fake news, online. The harmful effects of internet-based fake news extend beyond simply misleading audiences ^3^.

Intentionally fabricated and demonstrably false information, commonly known as fake news, presents a severe risk to democratic systems’ credibility. It undermines public trust in governmental institutions and significantly affects various societal sectors, such as elections, economic conditions, and public perceptions of critical issues such as wars ^4,5^.

**The aim of this research** stems from the urgent need to fight the increasing popularity of fake news, which is becoming increasingly common and significant on social media. Since fake news is so common, developing efficient detection systems is essential to maintaining the trust of online information.

Existing research indicates that fake news and authentic news exhibit notable superficial differences^6^. Fake news frequently leverages heightened emotional appeals and subjectivity^7^ , often incorporating phrases like “urgent notice” or “sharing quickly” to create a sense of urgency^8^. Additionally, images linked to fake news are usually of lower quality but are designed to be visually striking ^9^. On the other hand, genuine news tends to be objective, more detailed, and more accompanied by higher-quality visual imagery. Current multimodal approaches ^10–12^, which typically apply convolutional and recurrent neural networks (RNNs and CNNs), analyze these superficial characteristics by examining textual and visual components. Reports indicate that fake news attracts more attention on social media than factual news, a trend observable across major platforms ^13,14^. The high prevalence of fake news on social media presents a substantial challenge to the credibility of online information compared to other forms of misinformation. This pervasive issue underscores the urgent need to develop effective strategies to combat fake news. As data volumes grow, the rapid and efficient retrieval of relevant information becomes increasingly important. This highlights the critical role of computational linguistic techniques ^15^. Machine learning and deep learning methods are particularly crucial in this context, providing advanced tools to detect and counter misinformation effectively.

Machine learning and deep learning methods are particularly crucial in this context, providing advanced tools to detect and counter misinformation effectively. There is currently much interest in fake news identification because machine learning (ML), deep learning (DL), and natural language processing (NLP) have advanced recently, leading to the development of numerous innovative research methods ^16,17^.

E. Hashmi et al. raised doubts about the accuracy of the information that voters are exposed to during critical political events in their paper "Advancing Fake News Detection: Hybrid Deep Learning"^18^. They emphasized that during those times, more than 19 million bot accounts were made to disseminate erroneous information about Trump and Clinton, significantly increasing the amount of misinformation the public was exposed to and its impact ^19,20^. Furthermore, reports indicate that fake news frequently receives greater attention on social media platforms than true news, a pattern visible on several well-known sites ^21,22^. The reliability of online information is more seriously threatened by the widespread spread of fake news on social media than by other types of disinformation. Since fake news is a prevalent problem, creating efficient counterstrategies is becoming increasingly important.

The rapid and efficient retrieval of pertinent information is becoming increasingly important as the quantity of data continues to increase. This emphasizes how important it is to use computational linguistic approaches ^23^. Machine learning and deep learning techniques are essential because they provide advanced tools for effectively identifying and refuting misinformation.

Fake news identification has garnered increased attention due to recent developments in machine learning, deep learning, and natural language processing (NLP). These advances have given rise to many creative study methodologies ^24^. Because so much content is available online on a wide range of topics, this work becomes more complex, so researchers focus on creating automated techniques to detect fake news. Therefore, the integrity of internet information depends on this technological advancement ^25^. The identification of fake news is a significant technological obstacle due to multiple factors, necessitating sophisticated approaches to guarantee the authenticity and dependability of the information shared on the internet.

This paper employs ML and DL techniques, including cutting-edge transformer-based models, to improve fake news detection. We conduct a comprehensive and detailed analysis by incorporating fast text word embeddings for efficient text data processing and applying these methods to available datasets. This approach is vital for accurately identifying misinformation in online media.

The contributions of this paper are as follows:

1. It introduces a multilayer preprocessing framework that utilizes two sets of text datasets, one consisting of fake news and the other composed of real news.
2. The data preprocessing phase incorporates NLP techniques to prepare the data for use in word embedding.
3. We integrated both supervised and unsupervised FastText embeddings into machine learning (ML) models, such as decision tree (D.T), support vector machine (SVM), random forest (R.F), logistic regression (L.R), and extreme gradient boosting (XGBoost) bagging classifiers (CATBoost). We also devised a method to handle out-of-vocabulary words (OOV) using FastText embeddings so that our models may process phrases that have never been seen before, ensuring thorough coverage of text data. Furthermore, we diligently pursued optimization, optimizing regularization strategies and hyperparameters throughout our machine-learning models. With careful consideration, we hoped to maximize model performance, avoid overfitting, and generate reliable, broadly applicable findings.
4. In addition, we used FastText embeddings in DL-based models such as long short-term memory (LSTM), gated recurrent unit (GRU), and convolutional neural network (CNN) to efficiently capture intricate contextual information and sequential dependencies inside the text data. Additionally, this work employed the most recent transformer-based models for text categorization, such as the autoregressive transformer XLNET with hyperparameter tweaking, Robustly Optimized BERT (RoBERTa), and Bidirectional Encoder Representation from Transformers (BERT). We used these transformers because they have a track record of capturing complex contextual data and long-range correlations in text data, making them ideal for detecting fake news.
5. To enhance the interpretability of our results, particularly after observing the best performance of the hybrid model (Bi-LSTM–Bi-GRU), which integrates multiple deep learning models, achieved higher accuracy in classification than the other models.

**To achieve our goals for this research, we used several machine learning and deep learning techniques,** including state-of-the-art transformer-based models. In addition, we integrated FastText word embeddings with these models for effective text data processing using these techniques. We evaluated these models by conducting experiments on two publicly available Arabic datasets. Then, we compared the results obtained from these models and analyzed them to find the best model for detecting fake Arabic news. This method is essential for recognizing false information in online media, ultimately distributing more dependable and trustworthy information.

The organization of this paper is as follows: Section 2 reviews related research. Section 3 explains the proposed model architecture and methodology. Section 4 presents the results and insights, compares models within categories such as machine learning, deep learning, transformer models, ensemble learning, and hybrid models, and contrasts our results with those of other studies using the same dataset. The conclusions are presented in Sect. 5.

## Related work

In this section, we review the current literature on identifying fake news. Numerous studies have investigated various approaches, from transformer-based to conventional ML and DL techniques.

Han et al. ^26^ highlighted the robust modeling capabilities of pre-trained language models, exemplified by (bidirectional encoder representations from transformers) BERT. After extensive pretraining on corpora, these models have acquired significant syntactic and commonsense knowledge. Verma et al. ^27^ introduced word embedding over linguistic features for fake news detection (WELFake). This novel two-phase benchmark model authenticates news content by employing machine learning classification with word embedding over linguistic features. This comprehensive approach demonstrates a notable enhancement in fake news detection, with the WELFake model achieving a peak accuracy of 96.73%. This performance surpasses traditional methods such as BERT and CNN models by up to 4.25%, underscoring the effectiveness of integrating linguistic features with advanced embedding techniques. Additionally, the study contributes a novel dataset comprising approximately 72,000 articles, thereby bolstering the model’s reliability and generalizability across diverse datasets.

Shu et al. ^28^ introduced FakeNewsNet, a repository to facilitate research on fake news detection in social media. This repository includes two detailed datasets rich in news content, social context, and spatiotemporal information, aiming to overcome the limitations of existing datasets. The comprehensive Analysis of FakeNewsNet illuminates its potential applications in detecting fake news, addressing the challenges posed by the scarcity of multifaceted fake news datasets. This initiative represents a significant stride toward improving the accuracy and effectiveness of fake news detection mechanisms.

Truică and Apostol ^29^ introduced an innovative methodology utilizing document embeddings to develop multiple models that accurately classify news articles as trustworthy or fake. Their evaluation encompassed a range of machine learning (ML) models, including naive Bayes (N.B.), gradient boosting, deep learning (DL) models like long short-term memory (LSTM) and gated recurrent unit (GRU), as well as three transformer-based models: pre-trained BERT ^29^, (bidirectional and autoregressive transformers) BART ^30^, and RoBERTa ^31^.

These evaluations were conducted across five datasets containing fake news articles, employing various word embeddings such as TF-IDF, Word2Vec ^32^, and FastText ^33^. In a study by Nanade and Kumar ^34^, a transformer-based method utilizing the BERT base model for Twitter fake news detection achieved an accuracy of 77.29%. Verma et al. ^35^ introduced a binary classification framework for fake news detection that combines bidirectional encoder representations from transformers (BERT) to capture global text semantics and convolutional neural networks (CNN) to leverage N-gram features for local text semantics. Their experiments were conducted on four publicly available datasets. Guo et al. ^36^ proposed a similar approach using DL-based models and a pre-trained transformer-based BERT model for the same purpose. The results from both studies offer valuable insights into the effectiveness of these methods in fake news detection.

The study by Praseed et al. ^37^ focused on leveraging ensemble techniques with pre-trained transformer models such as XLM-RoBERTa ^38^, mBERT, and ELECTRA ^39^ to combat the proliferation of fake news, specifically in Hindi. The authors’ fine-tuning process tailored these models to discern misleading information across the linguistic nuances of Hindi effectively. This approach was validated on the CONSTRAINT dataset ^40^, which contains more than 8000 online posts delineated between nonhostile and hostile content, offering a nuanced understanding of the landscape of misinformation.

Wu et al. ^41^ introduced graph-based semantic structure mining with contrastive learning (GETRAL), a groundbreaking framework for semantic structure mining based on graphs coupled with contrastive learning. This innovation significantly enhances the identification of evidence-based fake news, outperforming existing models notably on the Snopes ^42^ and PolitiFact ^43^ datasets. By representing claims and evidence as graph-structured data, GETRAL effectively captures intricate semantic relationships, overcoming the limitations of previous methodologies. Graph structure learning reduces information redundancy and improves representation learning via supervised contrastive learning with adversarial augmented examples. On the Snopes dataset, GETRAL achieves an F1-Macro score of 80.61% and an F1-Micro score of 85.12%. On PolitiFact, it records an F1-Macro of 69.53% and an F1-Micro of 69.81%, demonstrating its superior performance in tackling the challenges of fake news detection by integrating advanced techniques for more precise and interpretable analysis.

Soga et al. ^44^ focused on detecting fake news on social media by analyzing stance similarity and employing graph neural networks (GNNs). Their approach revolves around assessing the opinion similarity between users based on their stances toward news articles and interactions within user posts. Leveraging graph transformer networks (GNNs), their method effectively extracts both global structural information and interactions of similar stances, addressing stance analysis challenges in microblogs while mitigating the impact of poorly represented stance features. Ying et al. ^45^ proposed a knowledge-enhanced semantic representation model named enhanced representation from knowledge integration (ERNIE), which shares structural similarities with BERT while leveraging multilayer transformers ^46,47^ as elemental encoders to model contextual information through self-attention mechanisms. Diverging from BERT, ERNIE incorporates semantic units such as words and entities, extending pretraining on word corpora abundant in knowledge to better model entity concepts and other semantic priors, enhancing the model’s semantic representation capabilities.

To lessen the effects of misinformation, especially in light of Russia’s aggression against Ukraine, Pilkevych et al. ^48^ investigated the detection of fake news using GNNs. They conducted a thorough analysis. They stress the use of GNNs in online media monitoring to quickly identify and evaluate fake news, suggesting that GNNs are powerful tools for the automated identification of damaging information.

Their method uses knowledge graphs (K.G.s) to map relationships and recognize entities in textual information, focusing on identifying indicators of harmful psychological influence. Among the models tested, GraphSAGE performed the best, attaining impressive accuracy scores of 98.01% on the Gossipcop dataset and 89.78% on the Politifact dataset when trained on data exhibiting indicators of detrimental psychological impact.

This study emphasizes how important it is to use advanced machine-learning approaches to combat misinformation. It also shows how GNNs may be used to improve the precision and efficacy of fake news detection systems.

Ying et al. ^49^ proposed enhanced representation from knowledge integration (ERNIE), a knowledge-enhanced semantic representation model that uses multilayer transformers ^50,51^ as the fundamental encoders for modeling contextual information through self-attention mechanisms. This model has a structure similar to that of BERT.

In contrast to BERT, ERNIE hides semantic units such as words and entities and expands pretraining on knowledge-rich word corpora. This enables improved modeling of previous semantic knowledge and entity concepts, ultimately improving the model’s capacity for semantic representation. In addition to being a context encoder for producing sentence expressions, ERNIE can also function as a knowledge repository, creating sentences implicitly from a large quantity of recorded factual knowledge. As a result, the ERNIE serves as the feature extractor of textual modalities, concurrently capturing the text’s surface and semantic properties. Dahou, Abdelghani, et al. ^52^ investigated how linguistic features—particularly Named Entity Recognition (NER)— identify false news. Two models were created: a token classification model for NER features and an AraBERT Multi-task Learning (MTL) model for identifying fake news. Machine learning techniques and an embedding fusion methodology were used to integrate the embedding vectors of these models. To improve performance, RLTTAO, a feature selection algorithm, was devised. It selects pertinent features. Findings indicated that adding NER features increased detection accuracy by 1.62% on average across 5 out of 7 datasets. Dahou, Abdelghani ^53^ used a Transformer model that has already been trained to extract characteristics from Arabic social media postings using several advanced approaches like Multi-task Learning (MTL). A customized Nutcracker Optimization Algorithm is used to enhance these properties. 87% for binary classification and 69% for multi-classification exhibit the framework’s high accuracy and beat traditional techniques. This helpful instrument gives the public trustworthy information, which also aids in the fight against fake news.

Alotaibi, Taghreed, and Hmood Al-Dossari ^54^ addressed that fake news detection in Arabic is less advanced than in English. It provided an overview of Arabic fake news research, detailing feature extraction methods, machine learning, and deep learning algorithms.

***The following are the present challenges in identifying fake news, based on the overview of the related work***:

1. Limited Research on Arabic fake news: Although the amount of work on detecting fake news is growing, there isn’t much research focusing just on Arabic-language content ^55^. This reflects a high demand for developing Arabic language solutions in this domain.

2. Emphasis on real-world applications: Although accuracy is the primary concern of numerous existing studies, research on the practical use of false news detection models in real-world scenarios is needed, taking user acceptability, scalability, and computational efficiency into account.

3. Explainable models are necessary: many current models, particularly those based on deep learning, are regarded as "black boxes." Confidence and spotting potential biases depend on knowing the rationale underlying model judgments.

4. Handling the changing of fake news: Since fake news strategies are ever-changing, it is necessary to have detection techniques that can adjust to new developments. Creating models that generalize well to many forms of fake news is crucial.

5. Variability and sophistication: Differentiating fake news from real news based only on outward appearances can be challenging because fake news frequently imitates real news in style and presentation. Misinformation strategies are becoming increasingly sophisticated and require sophisticated detection methods that can adjust to new patterns.

6. Linguistic nuances and Contextual Understanding: A thorough grasp of linguistic nuances and the capacity to perceive context are essential for successfully identifying fake news. The great range of languages and the unique cultural contexts in which news is distributed make this problematic.

7. Subjectivity and Bias: Finding biases and subjective claims in news articles is challenging without restricting free speech or adding biases to the detection process.

8. Scalability and Generalizability: Making detection algorithms scalable to handle enormous amounts of data on several platforms and generalizable to various subjects and languages is challenging. The literature currently in a publication makes clear that many researchers have addressed the issue of fake news identification using both conventional ML and DL-based algorithms, and they also emphasize the difficulties that the field is currently facing. These include the complex methods used to produce and spread false information, the speed at which false information spreads, and the challenge of detecting high levels of accuracy while preserving interpretability and generalizability.

So, our goal in this research is to expand the knowledge base in fake news detection for the Arabic language by focusing on Arabic news articles and developing language-specific models that can effectively address the unique challenges of Arabic text, such as complex linguistic structures and limited available resources. These models should be general enough to work on different Arabic datasets. These models include ML, transformer-based, and DL models. We integrate both supervised and unsupervised FastText word embeddings with these models to improve the generalizability and accuracy of fake news detection, along with various regularization strategies and hyperparameter tuning techniques.

## Proposed methodology

**Fig. 1:**
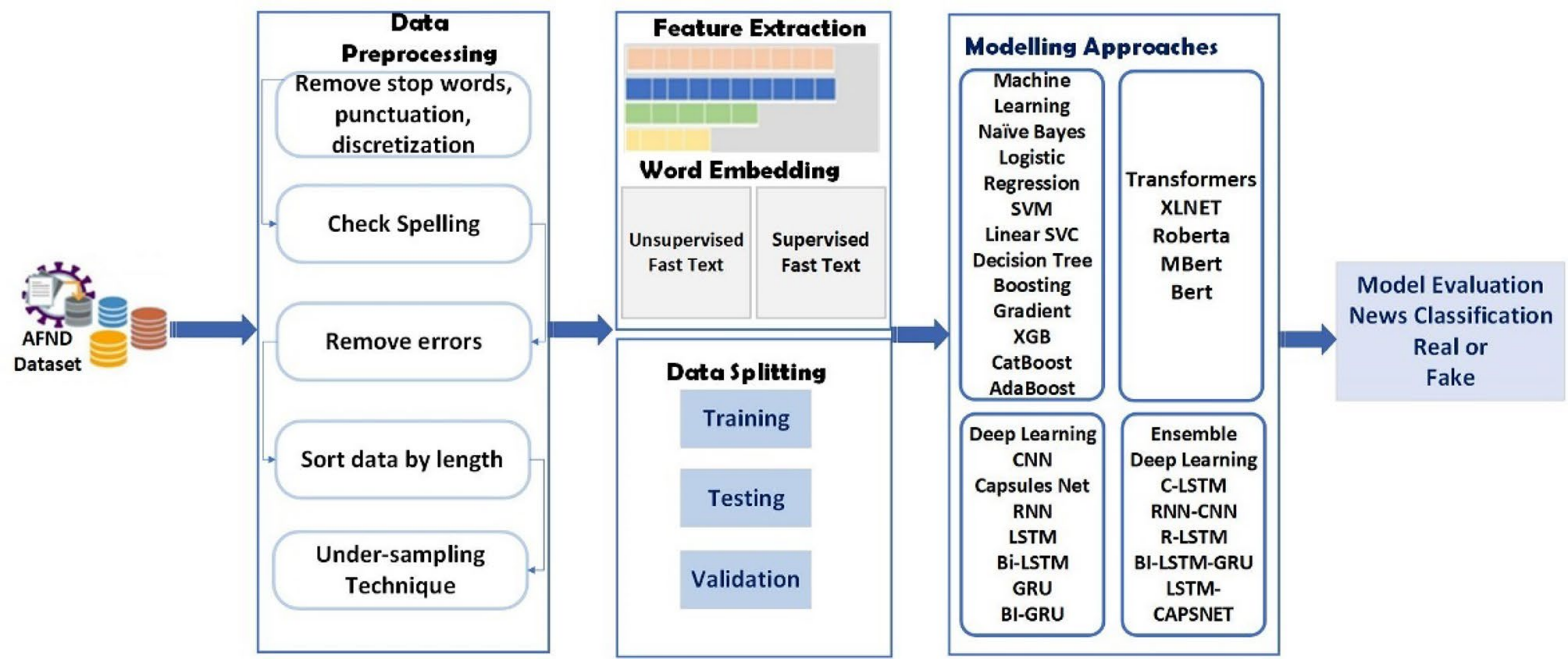
Methodology diagram for fake news detection.

In the proposed work, we propose different types of classifiers to identify the fake text. Then, we evaluate all these classifiers by conducting a set of experiments on two different Arabic datasets to find the best classifier to be used for detecting fake news in Arabic. With all these classifiers, the FastText library is applied to provide efficient word representations in order to enhance text classification. The proposed framework is shown in Fig. 1. As shown in Fig. 1, the proposed framework is comprised of three primary consecutive stages: the Data Collection and Preprocessing stage, the Textual Representation and Feature Extraction stage, and finally, the Modelling of Fake News Classifiers stage. The details of all these stages are explained next.

### A. Dataset collection and preprocessing stage

We adopted the binary classification problem in our study, where fake news is represented by 0 and real news by 1. Two publicly accessible datasets were utilized: The first dataset, AFND ^56^, comprises 134 distinct Arabic online news sources, providing a dataset of 606,912 Arabic articles. The dataset is divided into "real news," with 52 sources and 207,310 articles, and "fake news,” with 51 sources and 167,233 articles. The second dataset is Arabic Pocket Sheets ^57^. This dataset was created by acquiring 61,228 news sentences from an Arabic Twitter dataset (fake news) initially written in Arabic; Mendeley58 provided it. Web scraping was used to get 66,977 data instances from the well-known no-rumors (real news) website repository. We named this dataset “ARABICFAKETWEETS.” The details of both datasets are shown in Table 1.

**Table 1:** Number of instances in the used datasets.

Dataset	No. of fake articles	No. real articles	Total No. of articles
AFND	167,233	207,310	374,543
ARABICFAKETWEETS	61,228	66,977	128,205

Improving the performance of deep learning (DL) and machine learning (ML) models requires efficient data preprocessing. This process involves removing irrelevant text from the dataset and ensuring the data is formatted correctly and succinctly. Our paper focused on two key columns: "text," which included all the news comments, and "label," which identified the news as true or fake.

The primary purpose of text preprocessing is to significantly improve the effectiveness of learning algorithms. By carefully preparing the data, we can boost the quality and relevance of the information used for training and analysis. We execute crucial steps to preprocess the “text” column. The text undergoes several preprocessing steps before being fed into the classifier, significantly influencing the classification model’s overall performance.

First, we preprocessed the text by eliminating all non-Unicode letters and punctuation. After that, phrases are tokenized, stop words are eliminated, and tokens are drawn. By encoding the phrases into numerical sequences, we determine the unique token count and the maximum sentence length, ensuring that all the sentences are standardized to the same length. Labels are then encoded via one-hot encoding. This process utilizes the Arabic-Stop Words and Natural Language Toolkit (NLTK) ^59^ libraries. Stop words are removed through five different Python modules.

When dealing with high-dimensional data, dimensionality reduction techniques are essential for mitigating the risk of overfitting. The dataset is divided into two main groups, excluding uncertain news: “not credible” for fake news and “credible” for real news. The preprocessing operations include punctuation correction, tokenization, stemming, stop word removal, URL removal, and the elimination of names, all essential for refining the dataset.

We described the samples of AFND and ARABICFAKETWEETS datasets in Table 2 and Table 3, where in Table 2, we showed the original text before and after preprocessing for AFND Dataset, and in Table 3, we described the text before and after preprocessing for the ARABICFAKETWEETS dataset. Table 4 shows an English translation of samples taken from the Arabic Datasets.

**Table 2:** The text of AFND Dataset before and after preprocessing.

		S-No	Freq	Text	Label
The text of the AFND dataset	A. Before Preprocessing	1	254	{“articles”: [{“title”: “مجلة بلاي بوي الإباحية تشعر بالخجل من وضعها	Fake
		2	1499	{“articles”: [{“title”: “شاب متكبر يضع النفايات في سلة المهملات	Fake
		3	285	{“articles”: [{“title”: “أب يخير ابنه بين البربيش والخيزرانة ليعطيه مالا	Fake
		4	517	{“articles”: [{“title”: “الزعيم يوز ع المكرمات كاريكاتير محمد عفيفة	Fake
		5	425	{“articles”: [{“title”: “شاب يقاطع دكانا لم يعطه صاحبه البخيل كيس بيض	Fake
		6	379	{“articles”: [{“title”: “قرد يصاب بالقلق بعد سماعه إشاعات عن إمكانية تحوله لانسان	Fake
		7	616	{“articles”: [{“title”: “دراسة المواطن العربي يفني ربع عمره في البحث عن وظيفة	Fake
		8	639	{“articles”: [{“title”: “توق عات بارتفاع أرباح شركة الجامعة الأردنية العالمية	Fake
		9	1014	{“articles”: [{“title”: “ليبرمان النبي موسى أخطأ حين جاء بنا إلى الارض	Fake
		10	1254	{“articles”: [{“title”, “دليل أربع عقد نفسية تؤه لك لتصبح مديرا يطمح	Fake
	B. After Preprocessing	1	254	مجلة بلاي بوي الإباحية تشعر بالخجل من وضعها	Fake
		2	1499	شاب متكبر يضع النفايات في سلة المهملات	Fake
		3	285	أب يخير ابنه بين البربيش والخيزرانة ليعطيه مالا	Fake
		4	517	الزعيم يوز ع المكرمات كاريكاتير محمد عفيفة	Fake
		5	425	شاب يقاطع دكانا لم يعطه صاحبه البخيل كيس بيض	Fake
		6	379	قرد يصاب بالقلق بعد سماعه إشاعات عن إمكانية تحوله لانسان	Fake
		7	616	دراسة المواطن العربي يفني ربع عمره في البحث عن وظيفة	Fake
		8	639	توقعات بارتفاع أرباح شركة الجامعة الأردنية العالمية	Fake
		9	1014	ليبرمان النبي موسى أخطأ حين جاء بنا إلى الأرض	Fake
		10	1254	دليل أربع عقد نفسية تؤه لك لتصبح مديرا يطمح	Fake

**Table 3:** The text of ARABICFAKETWEETS dataset before and after preprocessing.

		S-No	Freq	Text	Label
The text of ARABICFAKETWEETS dataset	A. Before Preprocessing	1	211	صاحب سوبرماركت وضع لافته مضحكة على شفتيه press:	Fake
		2	145	\nRT @a_s_h1234567 شاب سعودى انقذ طفل كان يرضع من الامم المتحدة	Fake
		3	256	nRT @Amer_AlQah6ani: هبوط صاروخ باليستى بين مكة وجدة	Fake
		4	237	@qertyui5521 هزة ارضية بالمدينة المنورة تحدث انفجار ضخم	Fake
		5	345	@ALsiasi14\nRT السعودية تقرر منع تشغيل الموسيقى في المطاعم و المقاهي ب الرياض	Fake
		6	423	\nRT @Dr__Hussein غرامه وسجن عدم حمل البطاقة الشخصية فى اليمن	Fake
	B. After Preprocessing	1	211	صاحب سوبرماركت وضع لافته مضحكة على شفتيه	Fake
		2	145	شاب سعودى انقذ طفل كان يرضع من الامم المتحدة	Fake
		3	256	هبوط صاروخ باليستى بين مكة وجدة	Fake
		4	237	هزة ارضية بالمدينة المنورة تحدث انفجار ضخم	Fake
		5	345	السعودية تقرر منع تشغيل الموسيقى في المطاعم و المقاهي بالرياض	Fake
		6	423	غرامه وسجن عدم حمل البطاقة الشخصية فى اليمن	Fake

**Table 4:** The texts used in the dataset that were translated into the English language.

Text in Arabic	Text Translated into English
مجلة بلاي بوي الإباحية تشعر بالخجل من وضعها	Playboy porn magazine feels ashamed of its situation
شاب متكبر يضع النفايات في سلة المهملات	Arrogant Young Man Puts Trash in the Trash Bin
أب يخير ابنه بين البربيش والخيزرانة ليعطيه مالا	Father Gives His Son a Choice Between a Hose and a Stick to Give Him Money
الزعيم يوز ع المكرمات كاريكاتير محمد عفيفة	The Leader Distributes Gifts (Cartoon by Mohammed Afifa)
شاب يقاطع دكانا لم يعطه صاحبه البخيل كيس بيض	Young Man Boycotts a Shop Because the Stingy Owner Didn’t Give Him a Bag of Eggs
قرد يصاب بالقلق بعد سماعه إشاعات عن إمكانية تحوله لانسان	A monkey becomes anxious after hearing rumors about the possibility of him turning into a human.
دراسة المواطن العربي يفني ربع عمره في البحث عن وظيفة	Study: The Arab citizen spends a quarter of his life looking for a job
توقعات بارتفاع أرباح شركة الجامعة الأردنية العالمية	Expectations of an increase in the profits of the Jordanian International University Company
ليبرمان النبي موسى أخطأ حين جاء بنا إلى الأرض	Lieberman: The Prophet Moses made a mistake when he brought us to earth
دليل أربع عقد نفسية تؤه لك لتصبح مديرا يطمح	A guide to four psychological complexes that prepare you to become an aspiring manager
صاحب سوبرماركت وضع لافته مضحكة على شفتيه	A supermarket owner put a funny sign on his lips.
شاب سعودى انقذ طفل كان يرضع من الامم المتحدة	A young Saudi man saved a child who the United Nations was breastfeeding.
هبوط صاروخ باليستى بين مكة وجدة	A ballistic missile landed between Mecca and Jeddah.
هزة ارضية بالمدينة المنورة تحدث انفجار ضخم	An earthquake in Medina causes a massive explosion.
السعودية تقرر منع تشغيل الموسيقى في المطاعم و المقاهي بالرياض	Saudi Arabia has decided to ban music in restaurants and cafes in Riyadh.
غرامه وسجن عدم حمل البطاقة الشخصية فى اليمن	Fine and imprisonment for not carrying an I.D. card in Yemen

### B. Textual representation and feature extraction stage

Following data preprocessing and balancing, the subsequent stage focused on data representation and feature extraction, wherein the data were transformed into vector attributes for interpretation by machine learning (ML) and deep learning (DL) models. This process yields two types of features from the dataset: word embedding-based deep features and textual features, as elaborated upon in the following sections.

Word embeddings are methods that turn words into numerical vectors so that, when visualized, related words can be positioned next to one another. This technique captures words’ semantic and syntactic details from a large corpus, making sentiment analysis, entity recognition, and part-of-speech tagging applications valuable. Word2vec is a well-known pre-trained word embedding that provides word vector representations using two architectures: Continuous Skip-gram or continuous bag of words (CBOW). While skip-gram predicts surrounding words from a center word, CBOW predicts a word’s vector based on its surrounding words. Another widely used embedding, GloVe, constructs a co-occurrence matrix of words in a corpus and then factorizes this matrix to generate word vectors.

Choosing the right word embedding technique significantly impacts the performance of a deep learning model for detecting fake news in Arabic text. While both Word2Vec and GloVe are efficient and capable of capturing primary semantic relationships, they often struggle with context and polysemy issues, which are particularly problematic in Arabic fake news detection. These methods might not effectively differentiate between neutral and emotionally charged language or unusual word combinations, commonly used in fake news to manipulate readers.

Actually, the best word embedding method to be used for fake news detection classification is determined by many variables, including the target fake news’s features, computational capacity, and data accessibility. AraBERT-like contextual embeddings that have already been trained may be most suited for large dataset refinement. However, training these embeddings is computationally costly, which makes FastText a good starting option if resources are scarce. Another critical factor is the frequency of irony and sarcasm in the targeted fake news; contextual embeddings are probably necessary in these situations. To improve a deep learning model’s ability to identify fake news in Arabic, the best word embedding strategy can be found by carefully weighing these variables and trying various methods.

**Fig. 2:**
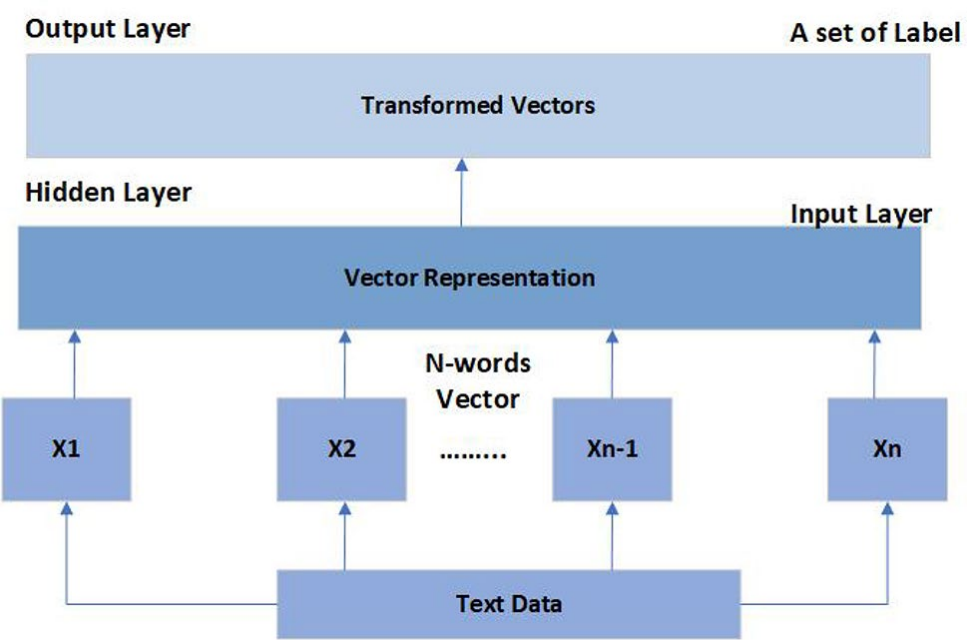
The word embedding architecture of FastText.

In this research, we adopted the FastText tool. FastText is a word representation tool developed by Facebook’s research team. FastText functions are in unsupervised and supervised modes. It boasts an extensive lexicon of 2 million words obtained from Common Crawl. Each word is embedded in a 300-dimensional vector space, resulting in a comprehensive library of 600-billion-word vectors. FastText architecture, as seen in Fig. 2, is an improvement over the Word2Vec CBOW model and provides additional benefits. It represents subword information and manages out-of-vocabulary words effectively, which is particularly advantageous for languages with complex morphological structures like Arabic language. Unlike traditional word vectors, FastText considers the internal structure of words, aiding in the representation of rare or misspelled words.

The mathematical formula for calculating FastText word embeddings is represented by Eq. 1 ^53^.

1 $$U_w+ \frac{1}{|N|}\sum_{\text{n }\in \text{ x}}{X}_{n}$$

Where:

U_w_ denotes the vector in the embedding space for a word w. This represents the vector in the embedding space for a given word www. It represents the word “w” as a vector of real numbers in high-dimensional space. Each word in the vocabulary has a corresponding vector.

$\frac{1}{|N|}$: is the fraction that represents the average.

∣N∣ denotes the number of context words around the target word “w.”

$\sum_{\text{n }\in \text{ x}}{X}_{n}$ : This refers to the summation of all vectors of the context words.

n ∈ N: denotes that we are adding up the values in the set N.

*X*_n_ denotes the vector of the set’s context words.

In our research, we adopted the use of FastText embeddings due to their proficiency in capturing semantic and contextual nuances in Arabic text data. These embeddings are supposed to enhance the performance of natural language processing tasks by providing numerical representations for textual inputs, enabling machines to process and comprehend textual data more efficiently. Moreover, FastText provides embeddings that consider subword information. This is essential for managing the intricate morphology of Arabic words, which differ greatly depending on their prefixes and suffixes. Furthermore, FastText better identifies slang and misspelled terms that are standard in fake or low-quality writing. However, if it is not adjusted for the particular task, it may bring noise from subword information.

Handling Out-of-Vocabulary (OOV) words in Arabic presents significant challenges due to the language’s rich morphology and dialectal variations^60^. To address this, we employ several strategies:

- **Subword-Level Embeddings (character-level n-grams):** by breaking down words into character sequences. For instance, the word " wrote" (books) is split into, "ك", "ت", "ب", "كت", "تب," "كتب".
- **Byte Pair Encoding (BPE)**: by learning subword units based on character pair frequencies. For instance, "الكتاب" (the book) might be split into "ال" and "كتاب."
- **Morphological analysis, including stemming and lemmatization:** by reducing words to their root form to increase vocabulary coverage; for example, "كتب" (wrote) is stemmed to "كتب" (write).
- **Hybrid Approaches**: combining subword and morphological techniques, capturing both subword and morphological information.
- **Out-of-Vocabulary Replacement:** It replaced OOV words with semantically similar words from the vocabulary, such as replacing "هاتف ذكي" (smartphone) with "جوال" (mobile phone) or initializing embeddings for OOV words randomly and fine-tuning them during training.

These strategies effectively manage the complexities of Arabic, improving the model’s performance in handling OOV words.

Our approach produces word embeddings for a Fake News dataset using FastText’s both unsupervised and supervised models to evaluate their efficiencies with the classification models.

In unsupervised learning, see **Algorithm 1**, FastText enhances the Word2Vec model by incorporating subword information through character n-grams (typically 3 to 6 characters). For instance, "الزعيم" “Leader” is broken down into fragments such as "زعي," "الز," and "عيم," while "المواطن" “Citizen” is dissected into "موا," "المو," and "اطن." This method aids in understanding word morphology and recognizing semantic connections between words with shared subparts. FastText utilizes extensive unlabeled text data to construct word representations, which can be applied to tasks such as word similarity and analogy.

**Algorithm 1:**
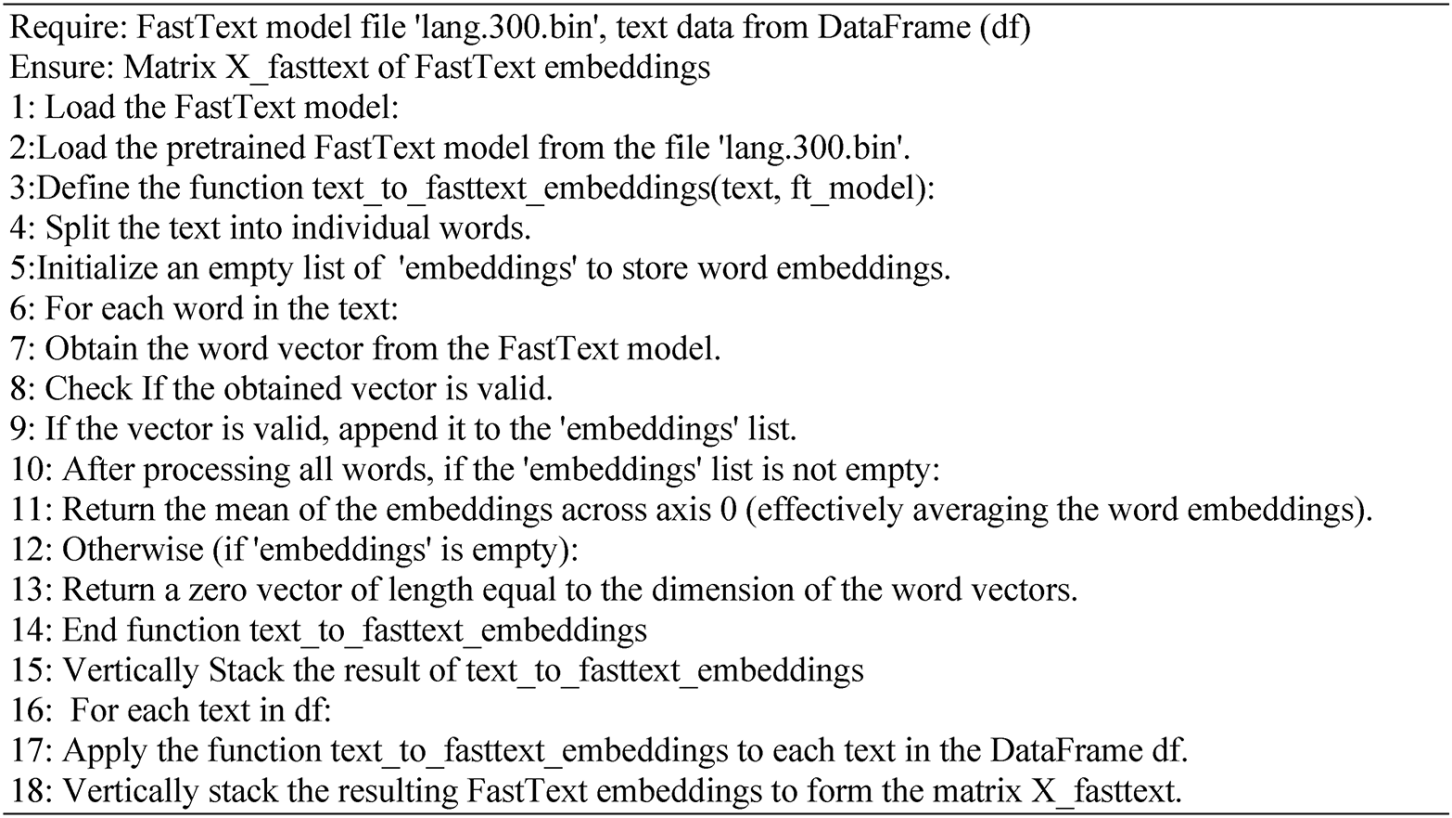
Unsupervised FastText embedding for text data.

In the supervised learning algorithm, see **Algorithm 2**, FastText is employed for text classification tasks. This algorithm incorporates subword information and trains on labeled datasets where each text excerpt is linked to a specific category or label. Using a hierarchical softmax function based on the Huffman coding tree, FastText accelerates training and prediction processes, efficiently handling large-scale datasets comprising millions of documents. During text classification, the model computes the average word vectors within a text to generate its representation, subsequently predicting its label. FastText’s supervised mode excels in managing extensive datasets and numerous classes.

Our research extensively explored both supervised and unsupervised FastText models and consistently observed superior performance with the supervised approach. This underscores the importance of labeled training data in text classification, where supervised learning leverages explicit category information to achieve heightened accuracy. Throughout our experiments, Through approximately 50 epochs, we trained the FastText model using different learning rates customized for the individual datasets. This strategic training regimen effectively harnesses the potential of FastText embeddings to enhance classification performance.

**Algorithm 2:**
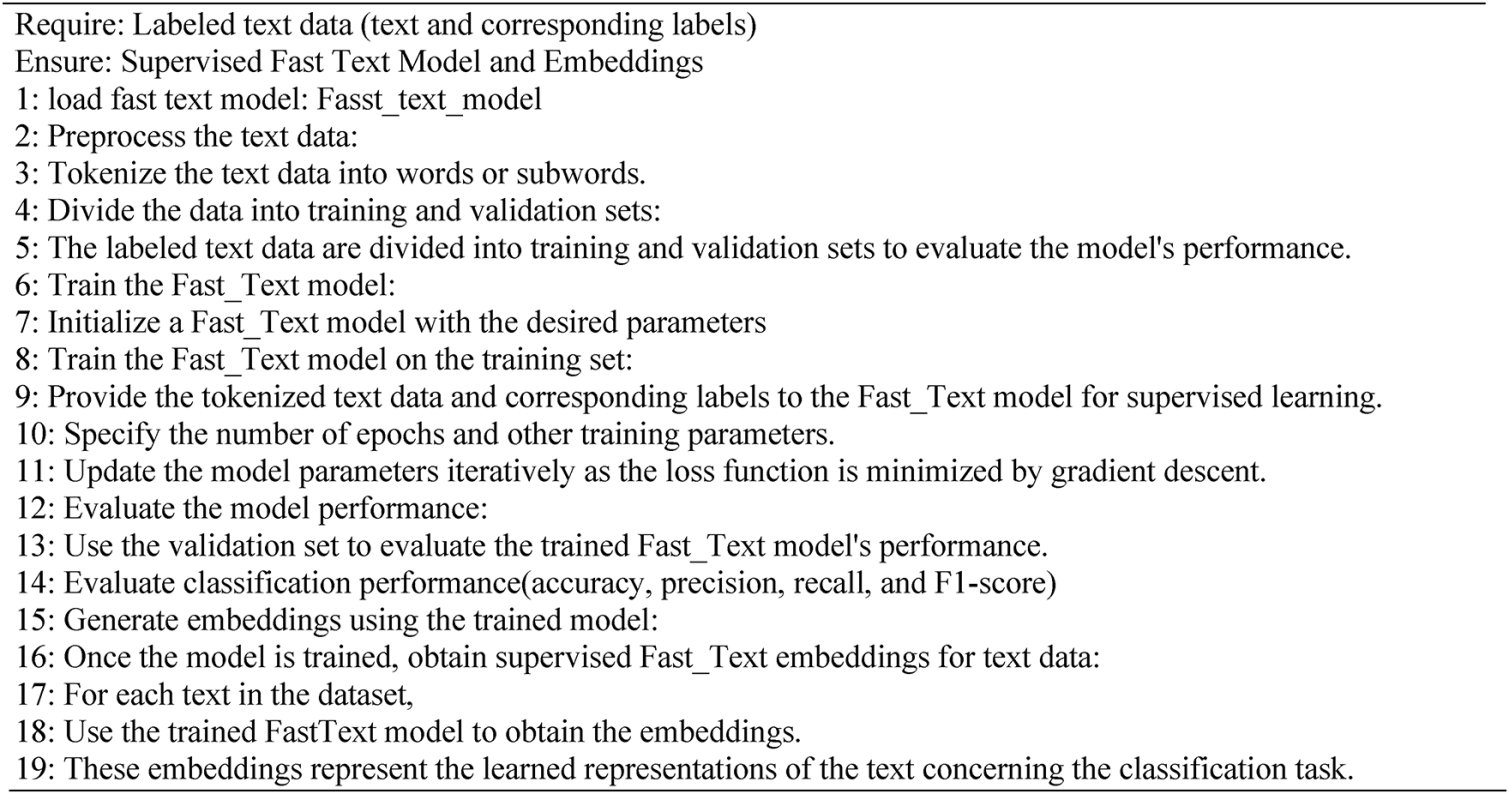
Supervised FastText embeddings for text data

### C. Modelling of fake news classifiers stage

This section comprehensively outlines first both the used machine learning (ML), boosting, and their hyperparameters configuration. Then, architectures of four different Ensemble deep learning classification models and deep learning (DL) thoroughly examine each model’s architecture and how it fits into our research approach. On the other hand, we introduce the hybrid Ensemble base techniques used to solve high-performance problems.

#### 1- Machine learning models

In our experiments, we used 5 common ML models. These include Naïve Bayes (N.B), Logistic Regression (L.R), Linear SVC, Random Forests (R.F), Support Vector Machines (SVM), and Decision Trees (D.T). In contrast, the boosting models used were Gradient Boosting, XGB, CatBoost, and AdaBoost. All these models employed FastText embeddings as input. Several regularization strategies and hyperparameters were implemented to maximize performance in these ML models, as explained next.

#### 1–1. Hyperparameter tuning for ML models

Table 5 lists the hyperparameters that can be utilized to optimize the machine-learning models. The parameter alpha is used with Naïve Bayes for smoothing; it is a positive constant added to the frequency counts of features to ensure that no probability is zero. The n-estimators parameter in the Random Forest (R.F.) model refers to the values that are used to test the model. The C parameter regulates the degree of regularization and helps to minimize overfitting of Logistic Regression (L.R.), Support Vector Machine (SVM), and Linear SVC classifiers. Lower values of C indicate more regularization, which limits the model to more superficial decision bounds. The parameter for ‘min_samples_split’ is used with the decision tree (D.T) classifier to represent the minimal number of samples required to divide a node, influencing the tree’s complexity and raising the risk of overfitting. GridSearchCV function can be used to automate the tuning of these hyperparameters.

**Table 5:** The configuration of Machine learning models.

Model	Regulation
Naïve Bayes	alpha: [0.1, 0.5, 1.0, 1.5, 2.0]
Logistic Regression	C: [0.01, 0.1, 1, 10, 100]
Linear SVC	C: [0.01, 0.1, 1, 10, 100],
Random Forest	n_estimators :[50, 100, 200]
SVM	C: [0.1, 1, 10, 100],
Decision Tree	‘min_samples_split’: ^2,5,9^,

#### 1–2. Hyperparameter tuning for boosting models

The hyperparameters utilized to improve different boosting models are listed in Table 6. Gradient Boosting used a subsample ratio of 0.7 to lessen the overfitting and computational load, a maximum depth of 3 to avoid overfitting while capturing complicated patterns, and a learning rate of 0.1 to strike a compromise between training speed and accuracy. To boost resistance, XGBoost (XGB) likewise employs 100 rounds and a learning rate of 0.1, but it also uses a slightly large subsample ratio of 0.7 and the same depth of 3. A deeper interaction set of five, a learning rate of 0.1, a subsample ratio of 0.7, and 100 boosting rounds are all included in CatBoost. On the other hand, AdaBoost employs 100 rounds and a far greater learning rate of 1.0, reflecting a more aggressive weight adjustment strategy.

**Table 6:** The configuration for boosting algorithms.

Model	Round	Learning rate	Depth	Sample
Gradient Boosting	100	0.1	3	0.7
XGB	100	0.1	3	0.7
CatBoost	100	0.1	5	0.7
AdaBoost	100	1.0	None	None

#### 2. Deep learning models

Deep learning models used in our research include:

- Transformers Models: These include the following common NLP transformer models:
- BERT (Bidirectional Encoder Representations from Transformers) is a transformer-based model for natural language understanding.
- RoBERTa (Robustly optimized BERT approach) is an improved variant of BERT with more training data and optimized hyperparameters.
- mBERT (Multilingual BERT) that extends BERT to support multiple languages
- XLNet is a generalized autoregressive pretraining method that outperforms BERT on several NLP tasks.
- Ensembles of common Deep Learning Models: These include some ensembles of the following common three DL models:
- CNN (Convolutional Neural Network) is commonly applied in text classification and NLP.
- RNN (Recurrent Neural Network) is designed for sequence data and time series, with variants like LSTM (Long Short-Term Memory) and Bi-LSTM (Bidirectional LSTM) addressing long-term dependency issues.
- GRU (Gated Recurrent Unit) is another RNN variant simplifying the LSTM architecture. Bi-GRU (Bidirectional GRU) processes data forward and backwards to capture context from both sequence ends.

In the following, we explain the architecture of each of the Ensemble models used in this paper.

#### 2–1 Ensemble-based models architectures

**Fig. 3:**
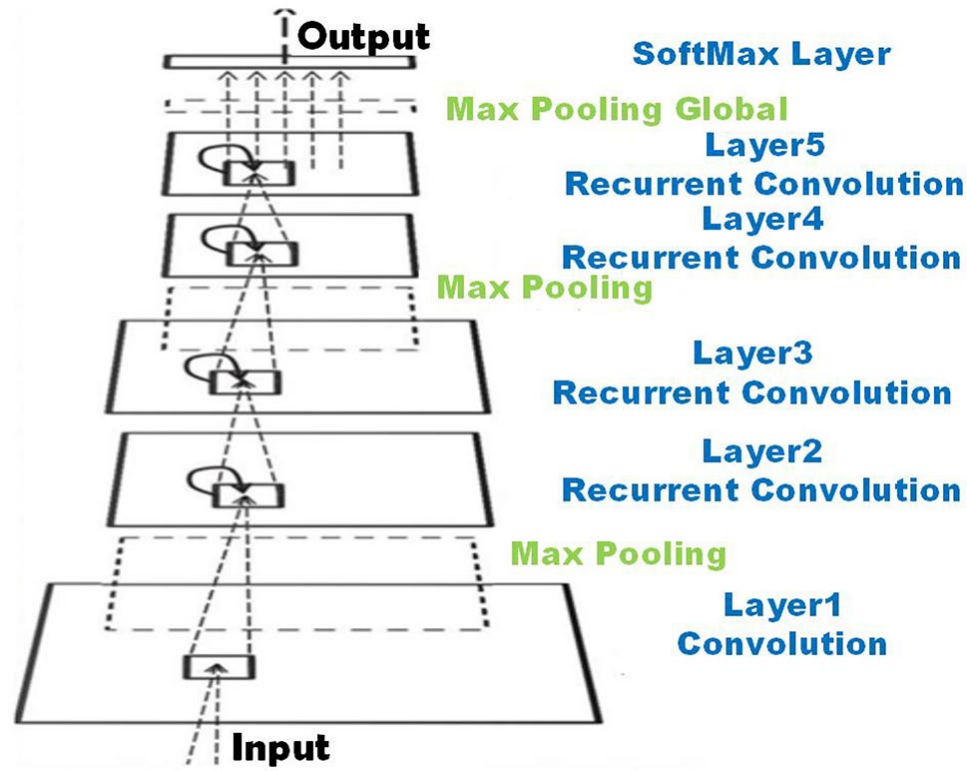
The hybrid model of RNN with CNN layers.

This paper examined four different Ensemble-based deep learning models, beginning with the RNN-CNN model. This model integrates a convolutional neural network (CNN) with four layers of recurrent neural networks (RNNs). The embedding layers’ outputs are initially processed through two traditional layers to extract features necessary for the subsequent RNN layers. The CNN layers apply max-pooling to the features and then concatenate them. In the final stage, a fully connected dense layer is utilized to predict the probability of each class label, as illustrated in Fig. 3.

**Fig. 4:**
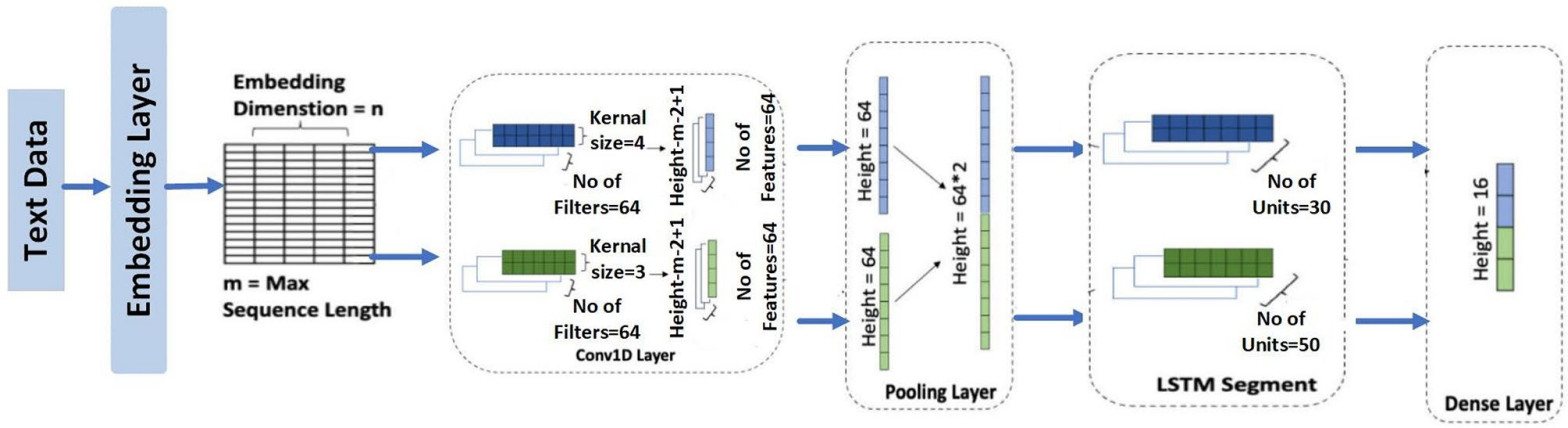
The hybrid model of LSTM with CNN layer.

A CNN-LSTM sequential deep learning architecture is the second model we used, and it was designed to identify Arabic Fake News (AFN) in particular. Because they can successfully manage different gap lengths, LSTM networks perform well in categorization and sequence processing tasks. As depicted in Fig. 4, our CNN-LSTM model starts with a convolutional layer that extracts features from the text embeddings. This layer uses 64 filters, each with a kernel size of 3. A max-pooling layer follows, reducing the dimensionality of the feature maps. Subsequently, an LSTM layer with 64 units and a dropout rate 0 processes these features as a time series, enabling the model to capture temporal dependencies in the data.

**Fig. 5:**
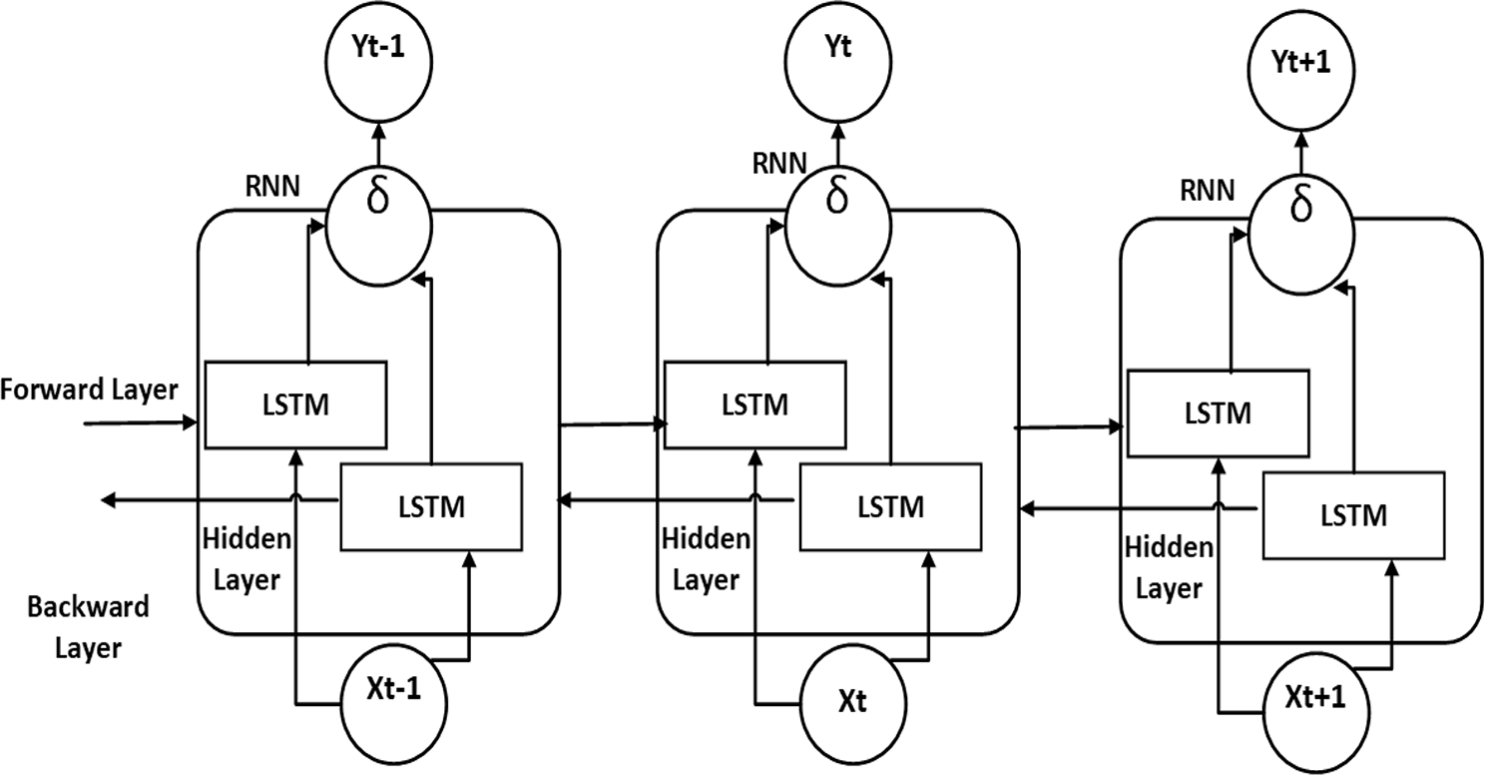
The hybrid model of LSTM with RNN .

The third model we explored is the RNN with LSTM model, illustrated in Fig. 5. This architecture integrates an embedding layer within a recurrent neural network (RNN) framework, connected to three LSTM layers and several one-dimensional convolutional layers with varying kernel sizes. These convolutional layers create diverse feature maps, enhancing the model’s interpretation of the data. The max pooling layers are paired with each convolutional layer to control feature compression and reduce overfitting. The outputs from these max-pooling layers are then merged using a concatenate layer.

The proposed model (hybrid architecture known as the Bi-LSTM-Bi-GRU) model, which combines the bidirectional gated recurrent unit (Bi-GRU) and bidirectional long short-term memory (Bi-LSTM) networks, has demonstrated promising results in the identification of fake news. By efficiently collecting long-term relationships and contextual information, this model uses the advantages of both Bi-LSTM and Bi-GRU to increase detection accuracy. Bi-LSTM networks are particularly effective at retaining information over long sequences due to their memory cell structures, which help maintain relevant context and address the vanishing gradient problem. In contrast, with their streamlined gating mechanisms, Bi-GRU networks provide computational efficiency and are well-suited for managing shorter dependencies. By integrating these two bidirectional models, the hybrid architecture processes input sequences in both forward and backward directions, ensuring a thorough understanding of the context. This dual-direction processing allows the model to identify intricate patterns and nuances in the text, which is essential for distinguishing between real news and misinformation. The methodology of the proposed model is presented in **Algorithm 3**.

**Fig. 6:**
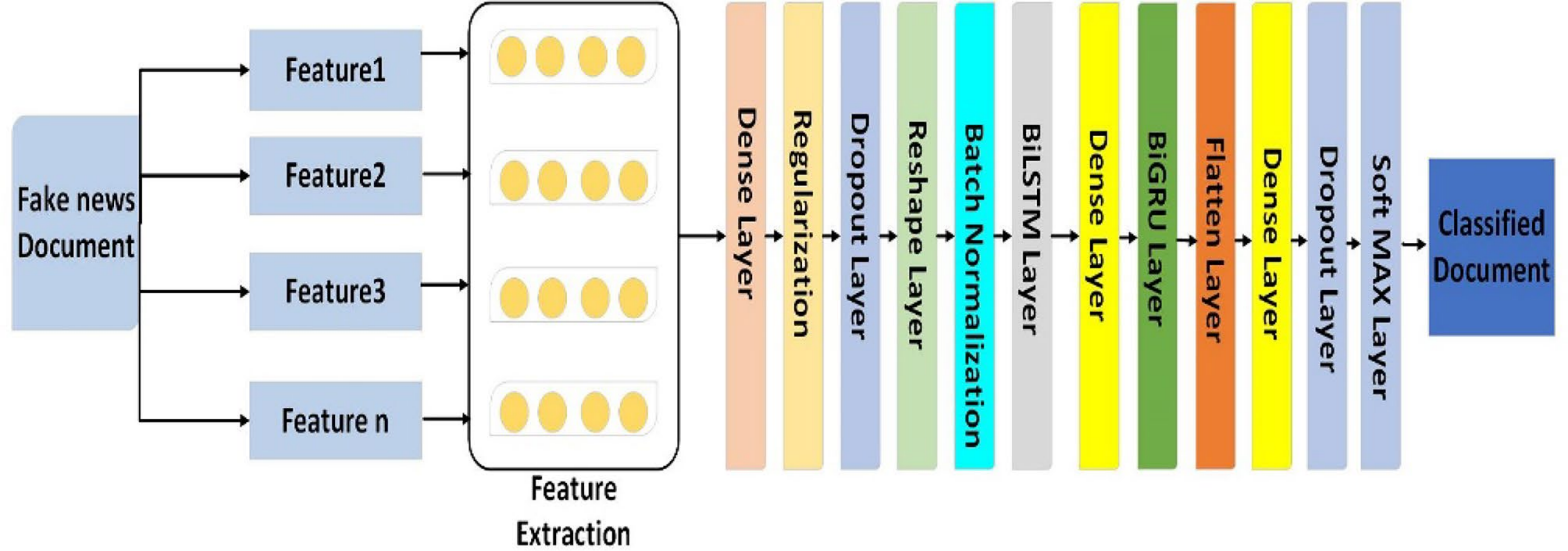
The proposed model of Bi-LSTM with Bi-GRU layers.

Figure 6 illustrates the primary architecture of the hybrid bidirectional LSTM (Bi-LSTM) and gated recurrent unit (GRU) (Bi-GRU) models. The neural network has nine layers and an input size of 200, corresponding to each word’s vector size. The model includes an embedding layer with a maximum input document length of 200 words, a vocabulary size of 5000, and an embedding dimension (EMBEDDING_DIM) 300. Both deep neural models in this hybrid architecture are 50-unit Bi-LSTMs with return_sequences set to TRUE and 50-unit Bi-GRUs with return_state and return_sequences set to TRUE. At the outputs of the Bi-LSTM and Bi-GRU models, global maximum pooling and global average pooling layers are introduced to improve the robustness against positional changes n features. The outputs from these pooling layers are concatenated to create a single feature vector. One dense layer that generates a single output is the model’s final output layer.

**Algorithm 3:**
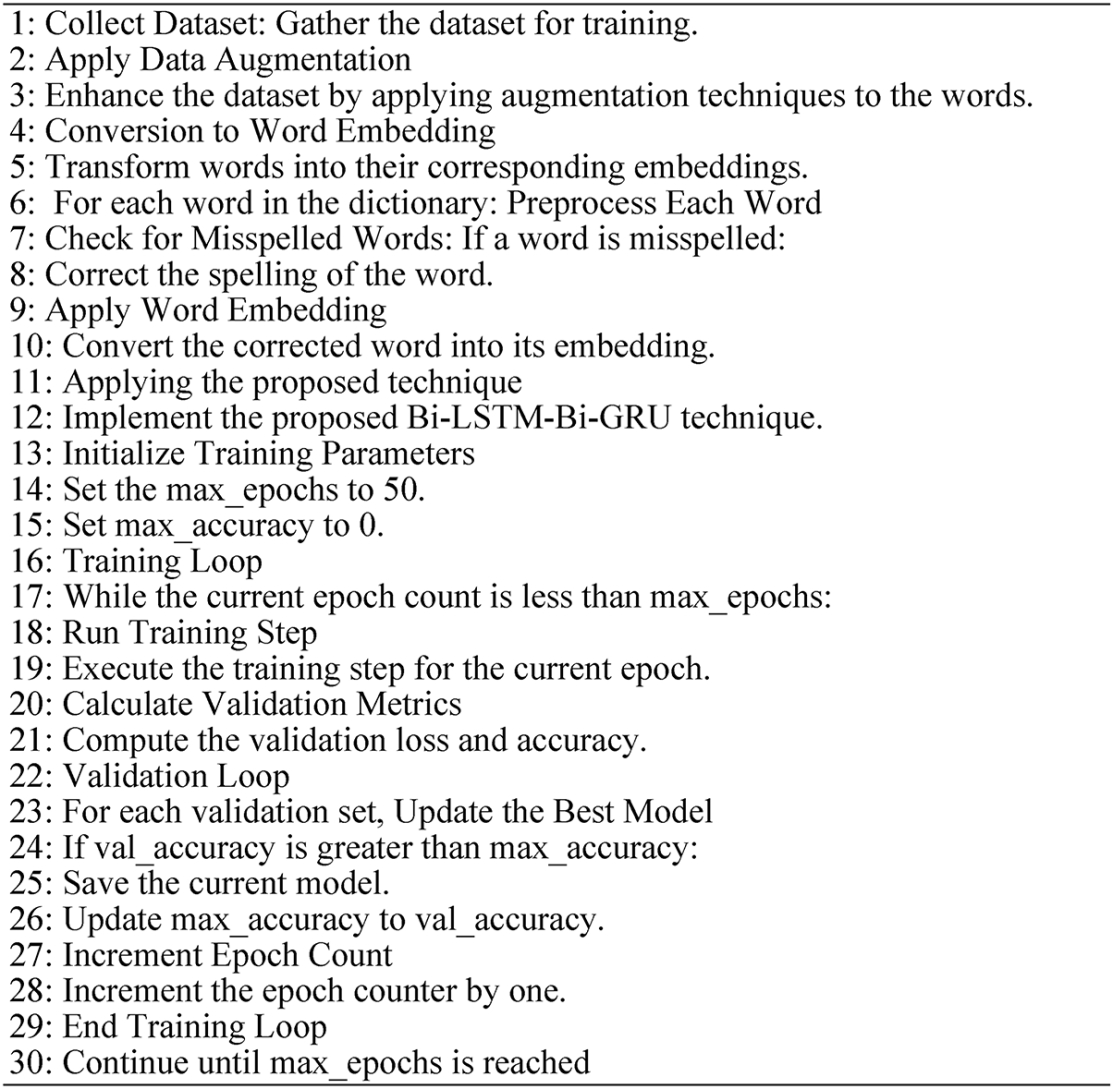
Bi-LSTM + Bi-GRU.

#### 2–2 Deep learning model configurations

The hyperparameter optimization process for deep learning (DL) involves carefully adjusting learning parameters through targeted experimentation. We adjusted the model’s learning process through targeted experimentation for both transformer-based and ensemble-based models.

#### 2–2-a. Hyperparameter tuning for transformer-based deep learning models

Table 7 lists the critical hyperparameter for transformer-based models. These include tokenizer, batch size, learning rate, and number of epochs—necessary for training a model. While MBERT, XLNET, and ROBERTa use AutoTokenizer to choose the best tokenizer for each model, BERT uses BertTokenizer to tokenize input text based on its pretraining. Tokenizers translate text into numerical values and tokens so that models can understand it. The batch size is 32 to balance computing efficiency and gradient update stability—a learning rate 2e-5 guarantees consistent convergence and moderate weight changes to prevent overshooting. The complete dataset is processed five times throughout the training process of the models, which allows for enough learning without overfitting.

**Table 7:** The configuration of the transformer-based models.

Model	Tokenizer	Batch	Learning rate	Epoch
BERT	BertTokenizer	32	2e-5	5
ROBERTa	Auto tokenizer	32	2e-5	5
MBERT	Auto tokenizer	32	2e-5	5
XLNET	Auto tokenizer	32	2e-5	5

#### 2–2-b Hyperparametertuning for ensemble-based deep learning models

The hyperparameters and configuration of each deep Ensemble-based learning-based model include the number of layers of each DL layer, the optimizer used in each model, the loss function, and the activation function used. For all the proposed deep leading models, the training time was set to 10 epochs to balance effective learning with the risk of overfitting, stopping when the model loss decreased. For example, the LSTM component, consisting of two layers with 50 and 30 units, captures temporal dynamics essential for analyzing sequential data. The model contains 2 layers for the model layer one dense layer and one dropout layer. The model includes a ‘softmax’-activated dense layer suitable for classification tasks. This architecture excels in extracting spatial features and understanding temporal sequences.

Table 8 describes how important hyperparameters and architectural components are optimized for hybrid neural network models. The RNN-CNN model has three layers: two dense, two dropout, two pooling, and one flatten. The CNN-LSTM and RNN-LSTM models were trained over ten epochs using the SoftMAX activation function, categorical cross-entropy loss, and the Adam optimizer. It consists of four layers: two dense, two dropout, two pooling, and one flatten. On the other hand, the Bi-LSTM-Bi-GRU model has two dropout layers, one flatten layer, three model layers—three dense layers—and no pooling layers. It trains for 10 epochs but uses the Relu activation function, with categorical cross-entropy loss and the Adam optimizer.

**Table 8:** The configuration of the ensemble-based deep learninmg models.

Model	Model layer	Dense layer	Dropout layer	Pooling layer	Flatten layer	Epoch	Function	loss	Optimizer
RNN-CNN	3	2	2	2	1	10	SoftMAX	Categorical entropy	Adam
CNN-LSTM	4	2	2	2	1	10	SoftMAX		
RNN-LSTM	4	2	2	2	1	10	SoftMAX		
BI-LSTM-Bi-GRU	3	3	2	0	1	10	Relu		

## Results and evaluations

To evaluate the fake detection models developed in this research, we conducted experiments on both the AFND Dataset and the ARABICFAKETWEETS Dataset. All the models have been developed using Python programming Tensorflow and Keras libraries. To run them, we used a Windows10–based machine with core i7 and 16 GB RAM. For te evaluation, we used metrics such as accuracy, precision, recall, and F1-score to gauge a model’s efficacy in differentiating between positive and negative instances. Then, we performed an Error analysis to assess the performance of the models by scrutinizing how well the models predict outcomes compared to actual data. Error analysis provides critical insights into where the machine learning models excel and where they falter, guiding improvements for enhanced predictive capabilities 53.

The formula for recall 54 is the ratio of true positives to the total number of false negatives and true positives. Precision 55 quantifies the proportion of accurate positive forecasts to the overall number of positive forecasts. The Accuracy 56 is a useful performance metric representing the percentage of accurately predicted observations out of all observations. The F1 score^55^ can be obtained from the area under the precision-recall curve and includes the effects of precision and recall. It is computed as the harmonic mean of these two values. This score provides an extensive evaluation of a model’s efficacy. All these metrics are easily calculated using the confusion matrix, which provides the values of True-positive (T.P), true-negative (T.N), false-positive (F.P), and false-negative (F.N) ^46^_._

These metrics are calculated for each model on both datasets two times. The first is when applying the unsupervised FastText embedding, and the second is when applying supervised FastText embedding. We evaluated all the obtained results to determine which model is better for detecting fake news. These results are presented next, followed by an error analysis. Then, a statistical significance analysis of the results will be conducted. Furthermore, an analysis of the models’ complexity is provided to assess their computational speed performance. Finally, a comparison with state-of-the-art fake news detectors is made.

### 1- Evaluation results of all models on the AFND dataset

Table 9 shows the results for the five ML models used in this paper: Naïve Bayes, Logistic Regression, Linear SVC, Decision Tree, Random Forest, and SVM. When using the unsupervised FastText, The Decision Tree model outperformed the other models as it obtained an accuracy of 0.77 and an F1 score of 0.76; in supervised FastText, the accuracy and F1 are 0.77 and 0.77, respectively.

**Table 9:** Results of supervised and unsupervised FastText with ML models on AFND dataset.

**Model**	**Unsupervised FastText**				**Supervised FastText**			
	**Precision**	**Recall**	**F1**	**Accuracy**	**Precision**	**Recall**	**F1**	**Accuracy**
Naïve Bayes	0.42	0.43	0.43	0.44	0.44	0.45	0.45	0.45
Logistic Regression	0.45	0.44	0.44	0.45	0.47	0.46	0.46	0.47
Linear SVC	0.33	0.33	0.33	0.34	0.35	035	0.35	0.36
**Decision Tree**	**0.74**	**0.74**	**0.76**	**0.77**	**0.77**	**0.75**	**0.77**	**0.77**
Random Forest	0.66	0.67	0.68	0.68	0.68	0.68	0.69	0.69
SVM	0.71	0.72	0.72	0.71	0.73	0.73	0.73	0.73

In Table 10, we show the results using the four boosting methods: Gradient Boosting, XGB, CatBoost, and AdaBoost. For most metrics, we observed that the Gradient-Boosting classifier achieved the best results. For the unsupervised FastText, the Gradient-Boosting classifier achieved a notable accuracy of 0.58 and an F1 of 0.60. In contrast, in Supervised FastText, the Gradient-Boosting classifier achieved 0.60 for accuracy and 0.60 for the F1 score.

**Table 10:** Results of supervised and unsupervised FastText with boosting models on AFND dataset.

**Model**	**Unsupervised FastText**				**Supervised FastText**			
	**Precision**	**Recall**	**F1**	**Accuracy**	**Precision**	**Recall**	**F1**	**Accuracy**
**Gradient Boosting Classifier**	**0.58**	**0.57**	**0.60**	**0.58**	0.56	**0.59**	**0.60**	**0.60**
XGB Classifier	0.53	0.54	0.53	0.53	0.56	0.56	0.55	0.55
CatBoost Classifier	0.45	0.43	0.46	0.44	0.47	0.46	0.48	0.46
AdaBoost Classifier	0.56	0.55	0.56	0.56	**0.58**	0.57	0.58	0.58

Table 11 presents the outcomes of the four Transformer-based models for the AFND dataset based on the number of epochs, which is 5 for each model during training; the XLNet transformer model achieves the best performance. With the unsupervised FastText, XLNet scored an accuracy of 0.87 and F1 of 0.86, while with supervised FastText, XLNet scored an F1 score of 0.88 and an accuracy of 0.88.

**Table 11:** Results of supervised and unsupervised FastText with transformer-based models on AFND dataset.

**Model**	**Unsupervised FastText**				**Supervised FastText**			
	**Precision**	**Recall**	**F1**	**Accuracy**	**Precision**	**Recall**	**F1**	**Accuracy**
Bert	0.65	0.64	0.66	0.66	0.67	0.66	0.68	0.68
RoBerta	0.77	0.76	0.78	0.79	0.79	0.78	0.80	0.80
MBert	0.80	0.80	0.80	0.81	0.82	0.82	0.82	0.82
**XlNet**	**0.86**	**0.86**	**0.86**	**0.87**	**0.88**	**0.88**	**0.88**	**0.88**

For both Unsupervised and Supervised FastText, Table 12 displays the outcomes of the six DL models: CNN, RNN, GRU, Bi-GRU, LSTM, and Bi-LSTM. In the case of unsupervised FastText, the Bi-GRU model has the highest performance, with 0.94 for an F1 score and 0.94 for accuracy after 10 epochs. Also, with the Supervised FastText, the Bi-GRU model was the best, with an F1 of 0.96 and an accuracy of 0.95 with 10 epochs.

**Table 12:** Results of supervised and unsupervised FastText with DL models on AFND dataset.

**Model**	**Unsupervised FastText**				**Supervised FastText**			
	**Precision**	**Recall**	**F1**	**Accuracy**	**Precision**	**Recall**	**F1**	**Accuracy**
CNN	0.52	0.52	0.53	0.53	0.54	0.54	0.55	0.55
RNN	0.82	0.83	0.83	0.83	0.84	0.85	0.85	0.85
GRU	0.93	0.93	0.93	0.93	0.94	0.93	0.95	0.95
**Bi-GRU**	**0.93**	**0.93**	**0.94**	**0.94**	**0.94**	**0.95**	**0.96**	**0.95**
LSTM	0.89	0.87	0.90	0.90	0.90	0.91	0.92	0.92
Bi-LSTM	0.91	0.91	0.92	0.92	0.93	0.93	0.94	0.94

Table 13 shows the results for the ensemble-based models. For unsupervised FastText, our proposed model (Bi-LSTM + Bi-GRU) outperformed all the other models in both Supervised and Unsupervised FastText embeddings. With an accuracy of 0.96 and an F1 score of 0.96 after 10 epochs. While in Supervised FastText, the proposed model (Bi-LSTM + Bi-GRU) achieved an F1 score of 0.98 and an accuracy of 0.98. This clearly indicated that the proposed model provides superior performance in comparison with all other models.

### 2- Evaluation results of all models on the ARABICFAKETWEETS dataset

**Table 13:** Results of supervised and unsupervised FastText with ensemble DL models on AFND dataset.

**Model**	**Unsupervised FastText**				**Supervised FastText**			
	**Precision**	**Recall**	**F1**	**Accuracy**	**Precision**	**Recall**	**F1**	**Accuracy**
RNN-CNN	0.82	0.82	0.83	0.83	0.84	0.84	0.84	0.85
CNN-LSTM	0.83	0.83	0.84	0.84	0.86	0.85	0.86	0.86
RNN-LSTM	0.86	0.86	0.87	0.87	0.89	0.89	0.89	0.89
**(Bi-LSTM + Bi-GRU)**	**0.95**	**0.95**	**0.96**	**0.96**	**0.97**	**0.97**	**0.98**	**0.98**

For the ARABICFAKETWEETS dataset, Table 14 displays the results for all the machine-learning models used in this paper. As shown in the table, the Decision Tree model obtains the best results with a high accuracy of 0.81 and an F1 score of 0.81 for Unsupervised FastText, 0.82 for F1, and 0.83 for accuracy in the case of Supervised FastText.

**Table 14:** Results of supervised and unsupervised FastText with ML models on ARABICFAKETWEETS dataset.

**Model**	**Unsupervised FastText**				**Supervised FastText**			
	**Precision**	**Recall**	**F1**	**Accuracy**	**Precision**	**Recall**	**F1**	**Accuracy**
Naïve Bayes	0.57	0.58	0.59	0.59	0.59	0.59	0.61	0.61
Logistic Regression	0.66	0.66	0.68	0.68	0.67	0.67	0.69	0.69
Linear SVC	0.46	0.47	0.48	0.48	0.48	0.48	0.50	0.51
**Decision Tree**	**0.80**	**0.80**	**0.81**	**0.81**	**0.81**	**0.81**	**0.82**	**0.83**
Random Forest	0.70	0.70	0.72	0.72	0.71	0.71	0.74	0.74
SVM	0.75	0.75	0.76	0.76	0.76	0.76	0.78	0.78

In Table 15, the results of applying boosting models on the ARABICFAKETWEETS Dataset are presented. We noticed that the Gradient Boosting model obtained all the highest scores, indicating its effectiveness in classifying defect news among the other machine learning models. It scored an accuracy of 0.72 and F1 of 0.73 in the case of Unsupervised FastText, and it scored F1 of 0.74 and accuracy of 0.74 when using Supervised FastText.

**Table 15:** Results of supervised and unsupervised FastText with boosting models on ARABICFAKETWEETS dataset.

**Model**	**Unsupervised FastText**				**Supervised FastText**			
	**Precision**	**Recall**	**F1**	**Accuracy**	**Precision**	**Recall**	**F1**	**Accuracy**
**Gradient Boosting**	**0.71**	**0.71**	**0.73**	**0.72**	**0.73**	**0.73**	**0.74**	**0.74**
XGB	0.65	0.65	0.66	0.66	0.67	0.67	0.68	0.68
CatBoost	0.58	0.58	0.59	0.59	0.59	0.59	0.61	0.61
AdaBoost	0.68	0.68	0.70	0.70	0.70	0.70	0.72	0.72

Table 16 summarizes the performance metrics for various Transformer-based models on the ARABICFAKETWEETS dataset. All models were trained for 5 epochs, indicating a consistent training duration. We noticed that XLNet achieved the highest performance with precision and recall at 0.92, an F1 score of 0.93, and an accuracy of 0.93 with Unsupervised FastText. With Supervised FastText, the XLNet model achieved 0.93 in both precision and recall and scored 0.94 for both F1 and accuracy.

**Table 16:** Results of supervised and unsupervised FastText with transformer models on ARABICFAKETWEETS dataset.

**Model**	**Unsupervised FastText**				**Supervised FastText**			
	**Precision**	**Recall**	**F1**	**Accuracy**	**Precision**	**Recall**	**F1**	**Accuracy**
BERT	0.77	0.77	0.78	0.78	0.78	0.78	0.79	0.79
RoBERTA	0.86	0.86	0.88	0.89	0.88	0.88	0.90	0.90
MBERT	0.89	0.89	0.90	0.90	0.91	0.91	0.92	0.92
XLNet	**0.92**	**0.92**	**0.93**	**0.93**	**0.93**	**0.93**	**0.94**	**0.94**

Table 17 presents the scored performance metrics for various deep learning techniques on the ARABICFAKETWEETS dataset, with all models trained for 10 epochs. With Unsupervised FastText, the Bi-GRU model achieved the highest performance, with a precision of 0.96, a recall of 0.95, an F1 score of 0.96, and an accuracy of 0.96. With Supervised FastText, the Bi-GRU model scored 0,97 for F1 score, 0.97 for accuracy, a precision of 0.96, and a recall of 0.96.

**Table 17:** Results of supervised and unsupervised FastText with DL models on ARABICFAKETWEETS dataset.

**Model**	**Unsupervised FastText**				**Supervised FastText**			
	**Precision**	**Recall**	**F1**	**Accuracy**	**Precision**	**Recall**	**F1**	**Accuracy**
CNN	0.61	0.62	0.63	0.63	0.63	0.63	0.64	0.64
RNN	0.88	0.88	0.89	0.89	0.90	0.90	0.91	0.91
GRU	0.94	0.94	0.95	0.95	0.95	0.95	0.96	0.96
**Bi-GRU**	**0.95**	**0.95**	**0.96**	**0.96**	**0.96**	**0.96**	**0.97**	**0.97**
LSTM	0.91	0.91	0.92	0.92	0.92	0.92	0.93	0.93
Bi-LSTM	0.93	0.93	0.94	0.94	0.94	0.94	0.95	0.95

Scored results for the Ensemble deep learning models on the ARABICFAKETWEETS dataset are shown in Table 18; all the models were trained for 10 epochs. The results show that the proposed model (Bi-LSTM + Bi-GRU) achieved the highest performance among all the other models; in Unsupervised FastText, it scored an F1 score of 0.98 and an accuracy of 0.98. In supervised FastText, it scored 0.99 for the F1 score and 0.99 for accuracy.

**Table 18:** Results of supervised and unsupervised FastText with ensemble DL models on ARABICFAKETWEETS.

Model	Unsupervised FastText				Supervised FastText			
	**Precision**	**Recall**	**F1**	**Accuracy**	**Precision**	**Recall**	**F1**	**Accuracy**
CNN-LSTM	0.91	0.91	0.92	0.92	0.92	0.92	0.93	0.93
RNN-CNN	0.88	0.88	0.88	0.89	090	0.90	0.91	0.91
RNN-LSTM	0.93	0.93	0.93	0.94	0.94	0.94	0.95	0.95
**(Bi-LSTM + Bi-GRU)**	**0.97**	**0.97**	**0.98**	**0.98**	**0.98**	**0.98**	**0.99**	**0.99**

**Fig. 7:**
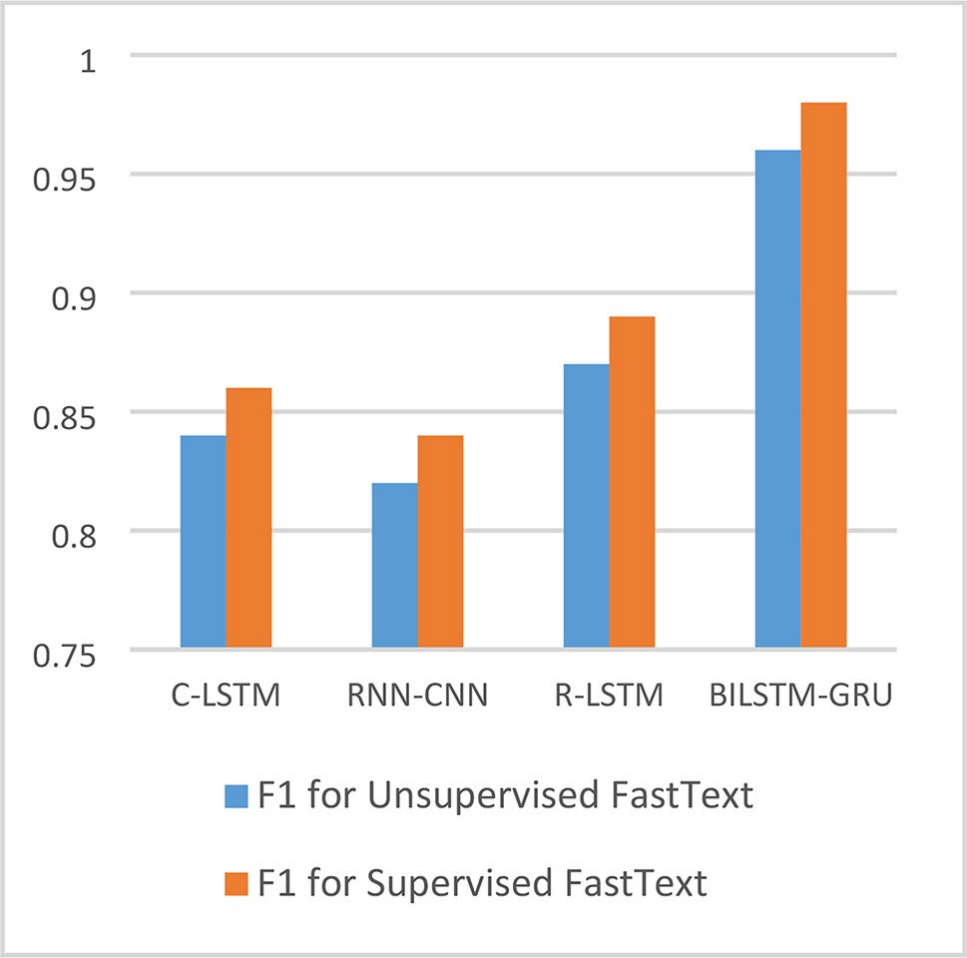
F1 for ensemble DL models for both unsupervised & supervised FastText on AFND dataset.

**Fig. 8:**
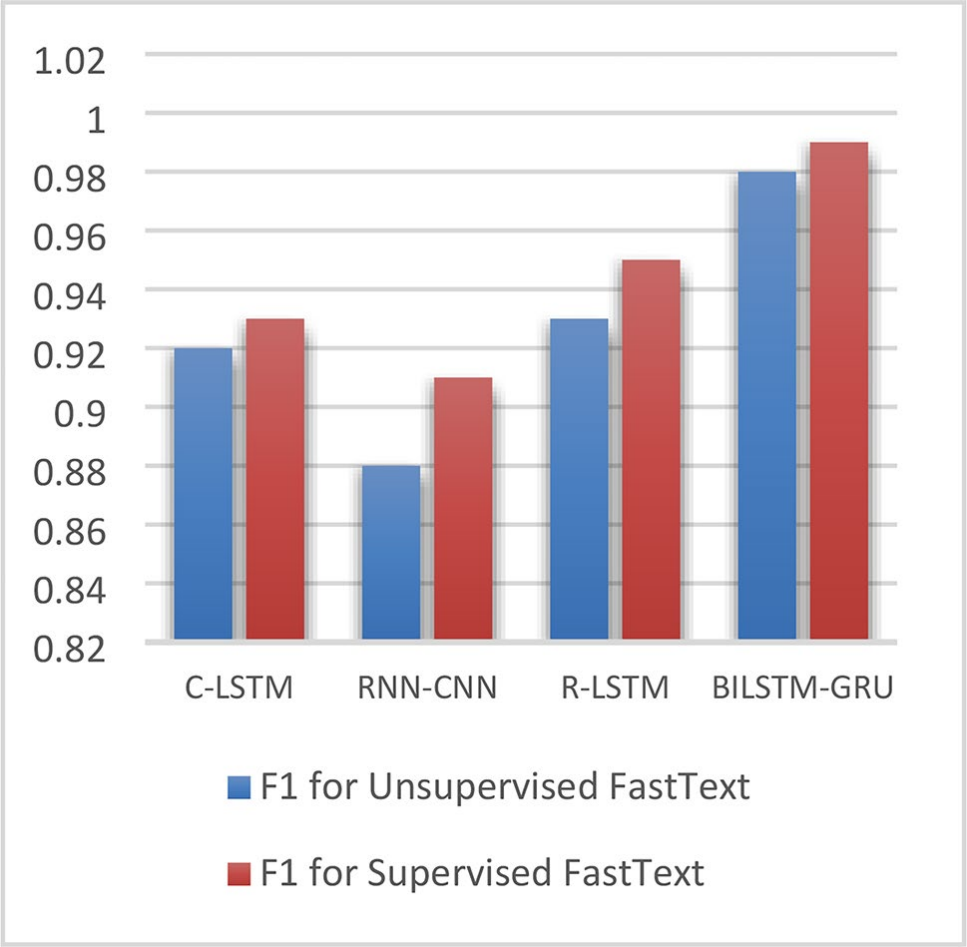
F1 for ensemble DL models for unsupervised FastText & supervised FastText on ARABICFAKETWEETS dataset.

Figures 7 and Fig. 8 show comparisons of F1-score values for the four ensemble DL models on both AFND and ARABICFAKETWEETS datasets. It is clearly seen that the proposed hybrid model Bi-LSTM + Bi-GRU outperforms the other models. It also shows that Using Supervised FastText enhances the performance significantly for all models.

**Fig. 9:**
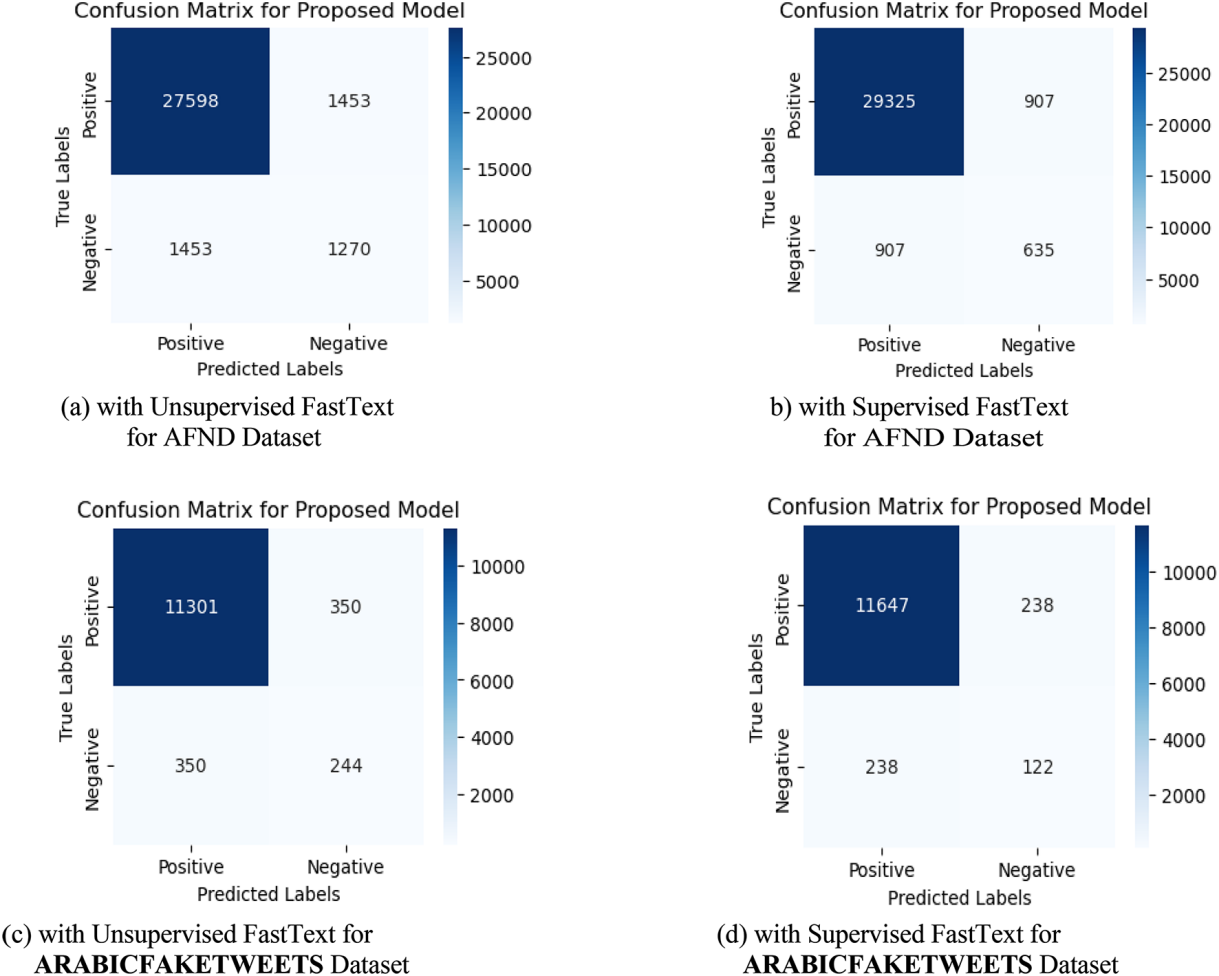
Confusion matrices of Bi-LSTM+Bi-GRU on both dataset.

Figure 9. shows the confusion matrices of our proposed model (Bi-LSTM + Bi-GRU) on both AFND and ARABICFAKETWEETS Datasets with both Unsupervised FastText and Supervised FastText.

From the above results of all models on both AFND and ARABICFAKETWEETS datasets, it is clearly seen that the proposed ensemble DL model (Bi-LSTM + Bi-GRU) with Supervised FastText scored the best results. In Fig. 7 and Fig. 8, we compared the four ensemble deep learning models in terms of the F1 score.

### 3- Error analysis

To understand the performance behavior of the models on the two datasets, we provide an error analysis of the misclassified instances of some of the most efficient classifiers. This analysis is based on the results obtained by running these models on the AFND dataset, which consists of 31,774 samples, and the ARABICFAKETWEETS dataset, which contains 12,245 samples, as explained next. Also, we analyze the features and attributes of the used Arabic samples on the efficiency of the proposed Bi-LSTM-Bi-GRU model that has the highest performance.

#### A- Error analysis of the misclassified instances with unsupervised FastText

For the AFND dataset, the results obtained when using Unsupervised FastText highlight significant differences in the performance of the models. When using the unsupervised FastText, we found that the RNN-CNN model, with an accuracy of 0.83, results in approximately 5,401 misclassified instances. The CNN-LSTM model, achieving an accuracy of 0.84, has around 5,086 misclassified instances. The RNN-LSTM model performed better, with an accuracy of 0.87, resulting in approximately 4,131 misclassified instances. The proposed Bi-LSTM-Bi-GRU model demonstrated superior performance with an accuracy of 0.96, resulting in only about 1,271 misclassified instances. This substantial improvement underscores the effectiveness of the Bi-LSTM-Bi-GRU architecture in accurately classifying instances within the dataset.

For the ARABICFAKETWEETS dataset. The CNN-LSTM model, with an accuracy of 0.92, results in approximately 970 misclassified instances. The RNN-CNN model, which achieves an accuracy of 0.89, has around 1,347 misclassified instances. The RNN-LSTM model performs better with an accuracy of 0.94, resulting in approximately 735 misclassified instances. The proposed Bi-LSTM-Bi-GRU model exhibited exceptional performance with an accuracy of 0.98, leading to only approximately 245 misclassified instances.

This significant enhancement highlights the effectiveness of the Bi-LSTM-Bi-GRU architecture in accurately classifying entries within the dataset. Compared to other models, the proposed Bi-LSTM-Bi-GRU model substantially reduced the error rate, demonstrating its robustness and suitability for more reliable real-world applications. This analysis indicated that the proposed model surpassed other models in terms of accuracy and significantly minimized misclassification, making it a more efficient option for this dataset.

#### B- Error analysis of the misclassified instances with supervised FastText.

For the AFND dataset, when using Supervised FastText, the CNN-LSTM model reported an accuracy of 0.86, which results in approximately 4,447 misclassified instances. RNN-CNN model, with an accuracy of 0.85, has around 4,766 misclassified instances. The RNN-LSTM model performs better, achieving an accuracy of 0.89 and leading to approximately 3,494 misclassified instances. These figures suggest that while CNN-LSTM and RNN-CNN have comparable performance, RNN-LSTM significantly reduces the number of errors, making it a more reliable choice. The proposed Bi-LSTM-Bi-GRU model, which integrates Bi-LSTM and Bi-GRU, demonstrates superior performance with an accuracy of 0.98, resulting in only around 635 misclassified instances.

For the supervised ARABICFAKETWEETS dataset, When using Supervised FastText, a significant enhancement occurs in the model performance. CNN-LSTM model, with an accuracy of 0.93, results in approximately 849 misclassified instances. RNN-CNN model, achieving an accuracy of 0.91, which leads to only around 1,097 misclassified instances. The RNN-LSTM model performs better with an accuracy of 0.95, leading to approximately 612 misclassified instances. These figures indicate that while the CNN-LSTM and RNN-CNN models have relatively higher error rates, the RNN-LSTM model notably improves misclassification. The proposed model, combining Bi-LSTM and Bi-GRU, demonstrates exceptional performance with an accuracy of 0.99, resulting in only about 122 misclassified instances. This significant improvement underscores the effectiveness of the Bi-LSTM + Bi-GRU architecture in accurately classifying instances within the dataset.

#### C- Error Analysis of the proposed (Bi-LSTM + Bi-GRU) model due to the nature of the dataset samples

Despite the superior performance of the proposed model (Bi-LSTM + Bi-GRU) in classifying tweets, some misclassifications still occur due to several inherent challenges. One primary reason is the ambiguity and overlapping features within the tweets themselves. Fake and real news tweets often share similar linguistic characteristics, making it difficult for the model to differentiate between them accurately. Additionally, tweets frequently contain informal language, slang, abbreviations, and emojis, which introduce noise and can confuse the model. Furthermore, errors in the labeled training data, where fake tweets are incorrectly labeled as real and vice versa, can also contribute to misclassifications.

Another significant factor is the Arabic language’s complexity and the tweet content’s diversity. Arabic tweets can vary widely in dialect, spelling, and colloquial expressions, challenging the model. Tweets that employ sarcasm, irony, or emotionally charged language are particularly difficult to classify correctly, as the literal content may be misleading. Moreover, the dynamic nature of news, where what is true at one point can be considered false later, adds to the difficulty. If the training data is not up-to-date or lacks comprehensive coverage of different topics and writing styles, the model may struggle with tweets that deviate from the patterns it learned during training. Addressing these challenges through improved data preprocessing, enhancing the diversity and quality of training data, and incorporating techniques to handle language nuances can help further reduce the number of misclassified instances.

###  4- Statistical significance analysis of the results

Because the Supervised FastText techniques outperformed unsupervised FastText techniques, we used a statistical significance analysis ^58^ for supervised FastText techniques for the two datasets, AFND and ARABICFAKETWEETS. Under the framework of the t-test, the null hypothesis (H0) posited that there would be no discernible difference in performance between the ensemble model and other hybrid DL models. Conversely, the alternative hypothesis (H1) asserted that there would indeed be a statistically significant divergence in accuracy between the proposed model Bi-LSTM + Bi-GRU and every model of the Hybrid DL models.

The statistical significance analysis for the AFND and ARABICFAKETWEETS datasets using a paired t-test reveals that the proposed Bi-LSTM + Bi-GRU model significantly outperformed other classifiers. For the AFND dataset, comparisons with CNN-LSTM, RNN-CNN, and RNN-LSTM yielded t-statistics of 24.76, 26.48, and 19.05, respectively, with p-values less than 0.0001. This indicated that the proposed model’s superior accuracy is statistically significant compared to these baseline models, highlighting its effectiveness in classifying data accurately. Similarly, in the ARABICFAKETWEETS dataset, the proposed model also demonstrated substantial improvements over CNN-LSTM, RNN-CNN, and RNN-LSTM, with t-statistics of 11.33, 16.70, and 10.81, respectively, and p-values below 0.0001. These results confirmed that the Bi-LSTM + Bi-GRU model achieved markedly higher accuracy than the other classifiers, underlining its strong performance across different datasets. The consistently low p-values across all comparisons reinforced the robustness of the proposed model’s advantage.

### 5- Comparison with the state-of-the-Art methods

In this section, we evaluate the proposed Bi-LSTM-Bi-GRU model that achieved the highest performance in this paper compared to other state-of-the-art models of Arabic fake news detection. For a fair comparison, we make comparisons with models that work on AFND and ARABICFAKETWEETS Datasets that are used in this paper.

#### A- Comparison with models working on the AFND dataset

Table 19. shows a comparison of our proposed Bi-LSTM + Bi-GRU model using Supervised FastText with CAPSNET^59^ and CNN-LSTM^60^. The table indicates that the proposed model outperforms the other models significantly by obtaining an F1 score of 0.98, an accuracy of 0.98, a recall of 0.97, and a precision of 0.97. This means that our work outperforms current state-of-the-art models and attains excellent performance.

**Table 19:** Comparison between our proposed model (Bi-LSTM + Bi-GRU**)** and other models on the same dataset (AFND).

Model	Precision	Recall	F1	Accuracy
CAPSNET ^59^	0.77	0.76	0.78	0.78
CNN-LSTM ^60^	0.80	0.80	0.81	0.81
Bi-LSTM + Bi-GRU	**0.97**	**0.97**	**0.98**	**0.98**

#### B- Comparison with models working on the ARABICFAKETWEETS dataset

For the ARABICFAKETWEETS dataset, we compared our proposed model (Bi-LSTM + Bi-GRU) that uses supervised FastText with the state-of-the-art ARBERT^55^ model^.^ The results are shown in Table 20. The results show clearly that our model (Bi-LSTM + Bi-GRU) outperformed the ARBERT model by achieving 0.99 for the F1 score and 0.99 for accuracy, but the ARBERT outperformed our proposed model in precision by scoring 0.99 while our model is scored slightly less precision value of 0.98.

**Table 20:** Comparison of (Bi-LSTM + Bi-GRU) with ARBERT model on ARABICFAKETWEET dataset.

Model	Precision	Recall	F1	Accuracy
ARBERT ^55^	0.99	098	0.98	0.98
**Bi-LSTM + Bi-GRU**	**0.98**	**0.98**	**0.99**	**0.99**

### 6- The evaluation of the computational time and space of the Bi-LSTM + Bi-GRU model

In the classification performance evaluation, it was shown clearly that the ensembled models achieved superior performance in comparison with other machine learning and deep learning models. Moreover, the proposed model Bi-LSTM + Bi-GRU showed the best results. In this section, we evaluate the computational efficiency of these ensembled models by comparing their time and space complexity. In general, the complexity of a combined model like RNN-CNN, CNN-LSTM, RNN-LSTM, and BiLSTM-GRU is computed by summing the complexities of its components^52^. Hence, for RNN-CNN, the time complexity is O(n*m*d^2^) + O (n*k*d*f^2^), where n is the sequence length, m is the number of samples, d is the hidden state size, k is the number of filters, and f is the filter size. The space complexity is O(n*m*d) + O(n*k*d*f). Also, For CNN-LSTM, the time complexity is O(n*k*d*f^2^) + O(n*m*d^2^) and the space complexity is O(n*k*d*f) + (n*m*d). For RNN-LSTM, the time complexity is O(2*n*m*d^2^), and the space complexity is O(2*n*m*d).

For the proposed model, Bi-LSTM-Bi-GRU, the time complexity is calculated by summing the of Bi-LSTM, which equals O(3*n*m*d^2^), and the time complexity of Bi-GRU, which equals O(n*m*d^2^) which yields a time complexity of O(3*n*m*d^2^). This means that d, which is the hidden state size, has the highest effect on the computation time, which is affected nonlinearly by its value. However, in comparison with the other hybrid models, we note that they all have the same relation with d as they all grow proportional to d^2^.

Also, for the space complexity, we add the space complexity of Bi-LSTM, which equals O(2*n*m*d), to the space complexity of Bi-GRU, which equals O(n*m*d ), yielding a space complexity of O(3*n*m*d). This means that the space complexity grows in a linear relation with n, m, and d. This relation is similar to the hybrid models mentioned above.

The above complexity analysis indicates that both the Time and Space complexity of the Bi-LSTM + Bi-GRU model is comparable with the other models in terms of the computing needs of time and memory for processing and storing data, activations, and model parameters. These intricacies aided in comprehending the viability and efficiency of implementing these models, particularly in the context of big datasets and real-time applications.

## Discussions

Our study’s findings show that the suggested Bi-LSTM + Bi-GRU model performs better than other models in identifying fake news in the AFND and ARABICFAKETWEETS datasets. Its high precision, recall, F1-score, and accuracy scores demonstrate the model’s effectiveness, which supports its potential for real-world use in fake news identification. Consistent performance across several datasets highlights the model’s robustness and generalizability.

On the AFND and ARABICFAKETWEETS datasets, the proposed model, Bi-LSTM + Bi-GRU with supervised FastText, performed effectively, with accuracy, precision, recall, and F1-scores ranging from 0.97 to 0.99.

### Implications

When considering a broader comparison among different models, including traditional machine learning, ensemble learning models, deep learning models, and hybrid deep learning models, our proposed model consistently outperformed the others. Moreover, it showed noticeable performance in detecting fake news when compared with some state-of-the-art Arabic fake news detection models. This makes our proposed model a valuable reference for detecting fake news in Arab countries. This is an essential step toward enriching the field of Arabic fake news detection, which is still in its early stages and requires significant efforts to expand it.

### Limitations

Even with the remarkable outcomes of this research, there are a few possible drawbacks. The dataset’s specificity is one of the main issues. The model may not perform well on some text kinds or themes if the datasets do not represent the Arabic text corpus. The complexity and variety of Arabic dialects present a significant challenge. Arabic contains multiple dialects that can differ significantly from Modern Standard Arabic (MSA), the language that is typically used in written documents. Arabic is not a homogenous language. While the model may work well on MSA, it may not be as generalizable or effective in other contexts due to its difficulties with regional dialects and informal language, which are common in social media. Another drawback is the rich morphology of the Arabic language, which allows words to take on a wide variety of forms through prefixes, suffixes, and infixes. This complexity makes tokenization and text preparation more difficult, which raises the possibility of mistakes affecting the model’s performance.

Furthermore, Arabic script is intrinsically ambiguous, with many words having several meanings depending on the context. This makes it challenging for the model to distinguish between different meanings correctly. For these linguistic difficulties to be overcome and to retain good performance across various datasets and real-world applications, more advanced preprocessing methods and resilient models that can handle the subtleties of the Arabic language are required.

The interpretability of the model is another limitation; deep learning models such as Bi-LSTM and Bi-GRU are frequently seen as "black boxes," making it challenging to comprehend how they make decisions. Such models usually require a lot of resources, so not all users may have access to sophisticated hardware.

### Future work

Future research should aim to create fresh approaches to improve the transparency and reliability of A.I. systems in identifying false news. Lastly, it’s essential to keep updating detection techniques as disinformation strategies change.

To ensure these models remain effective over time, future research should concentrate on modifying them to identify and react to novel forms of misinformation tactics and fake news. Future research can improve the results of this study by addressing these areas and help create more transparent, flexible, and successful fake news detection systems.

Improving the quality and diversity of the training data is crucial to addressing misclassification issues in future work. Expanding the dataset to include more diverse examples, such as tweets from different regions and contexts, will help the model generalize better. Employing advanced data augmentation techniques, like synonym replacement and back-translation, can generate new training examples. Additionally, ensuring the accuracy of labeled data through rigorous annotation processes and cross-verifying labels can significantly reduce errors.

Leveraging transfer learning from larger, pre-trained models can also enhance performance. Additionally, integrating external knowledge bases and considering the social context of tweets, such as user profiles and retweet patterns, can provide valuable context for verifying information. Implementing online learning techniques to keep the model current with evolving language and trends and employing active learning to iteratively improve the model by focusing on challenging examples can ensure continuous improvement and adaptation.

The results of this study can be expanded by examining many vital topics. Text combined with multimodal data (pictures and videos, for example) may improve the detection of fake news even further. Subsequent research ought to investigate how merging disparate data kinds impacts model efficacy and discernment potential. To enhance the resilience and relevance of models for detecting false news, more studies should broaden the scope of datasets to encompass a greater variety of languages and cultural backgrounds.

This can assist in addressing the linguistic subtleties and cultural variances in fake news. A useful development would be creating real-time detection systems that can process and analyze information as it is distributed. Research should concentrate on speed and efficiency model optimization to manage massive volumes of data in real-time. More developments in XAI methods may also offer a more profound understanding of how complicated models make decisions.

## Conclusion

This study addresses the urgent problem of fake news spreading on social media by providing a thorough and reliable methodology for identifying fake news. We thoroughly assessed the effectiveness of FastText embeddings in conjunction with cutting-edge ML and DL-based methods using two different datasets, focusing on optimization tactics to prevent overfitting and address generalizability. We suggested that models leveraging deep learning and machine learning, particularly those utilizing text-based linguistic features in Arabic, effectively detect fake news. While no single traditional machine learning algorithm or boosting method consistently and significantly outperforms the others, ensemble deep learning techniques achieve high performance. This paper introduced a novel integration of FastText embeddings with advanced machine learning and deep learning models, specifically tailored to enhance the understanding and processing of Arabic linguistic features. We also developed and evaluated Four hybrid techniques to improve the performance of the fake news detection system. These hybrid models consistently demonstrated superior performance in fake news detection tasks: CLSTM (CNN + LSTM), RCNN (CNN + RNN), RLSTM (RNN + LSTM), and Bi-LSTM and Bi-GRU. Our results indicate that the hybrid model integrating Bi-LSTM with Bi-GRU layers, enhanced with FastText embeddings, outperforms significantly other models in accurately classifying news articles.

Additionally, transformer-based models highlight their ability to understand complex syntactic structures, enhancing semantic comprehension. Our proposed model, Bi-LSTM + Bi-GRU, exhibited strong performance beginning in the initial epochs, and its performance continued to improve with increasing epochs, ultimately achieving an impressive F1 score of 0.98 and 0.99 for the AFND dataset and ARABICFAKETWEETS dataset respectively. When considering a broader comparison among different models, including traditional machine learning, ensemble learning models, deep learning models, and hybrid deep learning models, our proposed model consistently outperformed the others. This makes our proposed model a valuable reference for detecting fake news in Arab countries. In future work, we aim to investigate many approaches to improve the application and effectiveness of the model. One possible approach to increase detection accuracy is to include more Arabic-specific linguistic elements, such as dialect variances and stylistic nuances. Performance enhancements may also be obtained using transfer learning from pre-trained language models explicitly adjusted on Arabic text corpora. Creating lightweight models that preserve high accuracy while being computationally efficient, allowing for deployment on portable and low-power devices, is another crucial field. Also, we aim to extend fake news detection to multiple languages by investigating the potential of multilingual transformers, such as mBERT, mT5, and GPT. Also, it seeks to make further progress in combating the global problem of fake news propagation by implementing adversarial training techniques.

## Data Availability

The datasets used and/or analyzed during the current study are available from the corresponding author upon reasonable request.
